# *Adiantum capillus‐veneris:* A Comprehensive Review of Its Medicinal Properties, Bioactive Compounds, and Advanced Extraction Techniques

**DOI:** 10.1002/fsn3.71118

**Published:** 2025-10-21

**Authors:** Syamand Ahmed Qadir, Farhang Hameed Awlqadr, Miran Hussein Qadir, Ako Mahmood Qadir, Aryan Mahmood Faraj, Salah Abdulla Salih, Mohammed N. Saeed, Khaled Arab, Seyed Mohammad Najibi Hosseini

**Affiliations:** ^1^ Medical Laboratory Techniques Department, Halabja Technical Institute, Research Center Sulaimani Polytechnic University Sulaymaniyah Iraq; ^2^ Food Science and Quality Control Halabja Technical College, Sulaimani Polytechnic University Sulaymaniyah Iraq; ^3^ Department of Food Science and Technology Faculty of Agriculture, University of Tabriz Tabriz Iran; ^4^ Department of Medical Laboratory Science College of Science, University of Raparin Sulaymaniyah Iraq; ^5^ Medical Laboratory Science Department Halabja Technical College, Sulaimani Polytechnic University Sulaymaniyah Iraq; ^6^ Medicinal Plant Department Halabja Technical College, Sulaimani Polytechnic University Sulaymaniyah Iraq; ^7^ Department of Nutritional Analysis and Health Kifri Technical College, Garmian Polytechnic University Kifri Sulaimaniyah Province Iraq

**Keywords:** *Adiantum capillus‐veneris*, *antidiabetic*, *antimicrobial*, *antioxidant*, *anti‐inflammatory*, *bioactive compounds*

## Abstract

Maidenhair fern ( *Adiantum capillus‐veneris* ) is a common medicinal plant used in traditional systems to treat various illnesses. Its rich phytochemical composition, which includes flavonoids, phenolic acids, triterpenoids, saponins, and tannins, is thought to be responsible for its medicinal qualities. The review illustrates the pharmacological activities of *Adiantum capillus‐veneris* , focusing on its antioxidant, anti‐inflammatory, antimicrobial, and antidiabetic potentials, as supported by in vitro and in vivo studies. Antioxidant activity is the main factor in the plant's success in clearing out the reactive oxygen species (ROS), as tested in the case of notable effects from DPPH. The relationship between inflammation and the capability of prospection and treatment of inflammatory mediators such as TNF‐α, IL‐6, and prostaglandin E2 is the manner in which this gathering of the NF‐κB pathway is conducted. Also, the plant possesses antidiabetic properties through the α‐glucosidase and other carbohydrate‐metabolizing enzymes. Cutting‐edge technology involving the use of advanced extraction and analytical methods, such as Soxhlet extraction and GC–MS has interacted with the identification of bioactive compounds. Even so, problems are not absent, as standardized extraction protocols and minimal clinical trial data are still scarce. Additionally, our understanding of molecular mechanisms is lacking. This paper presents *Adiantum capillus‐veneris* as a natural remedy with great promise. To ensure its effectiveness and safe incorporation into modern medicine, adequate investigation (such as clinical studies and formulation development) is essential.

## Graphical abstract

Adiantum capillus‐veneris (Maidenhair fern) is a medicinal plant known for its rich phytochemical composition and diverse therapeutic properties. This review highlights its antioxidant, anti‐inflammatory, antimicrobial, and antidiabetic activities, along with modern extraction techniques for isolating bioactive compounds. Despite promising evidence, further clinical research is essential to confirm its medicinal potential.

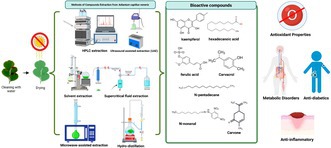

## 1 Introduction

Throughout the centuries, the role of medicinal plants in treating different medical conditions has been very significant (Manisha et al. 2025). Their therapeutic effects have drawn the interest of researchers worldwide, particularly regarding the treatment of prevalent conditions like atherosclerosis, cerebrovascular events, diabetes, hypertension, and Alzheimer's disease (Abdulkhader et al. 2023; Yadav 2021). In addition, the studies show that medicinal plants carry a long and diverse list of biological effects, among which are the antiviral, antifungal, antibacterial, antiproliferative, antimutagenic, anti‐inflammatory, antioxidant, anticancer, anticarcinogenic, and antidepressant effects (Lakshmi et al. 2021; Parham et al. 2020). Phytochemicals are also the natural compounds found in plants, and they have been the focus of research because of their potential for the development of novel therapeutic drugs. As opposed to synthetic drugs, phytochemicals can show comparable bioactivity with fewer side effects (Chauhan et al. 2019; Gorlenko et al. 2020). Furthermore, the increasing number of antibiotic‐resistant strains has shed more light on the need for plant‐derived biomolecules as the best weapon in the fight against resistant pathogens (Cappiello et al. 2020).

Plants are a rich source of medicinal substances, including flavonoids, carotenoids, and polyphenols that contribute to their antioxidant, cancer‐fighting, and disease‐preventing effects. Furthermore, the presence of some vital nonmetallic and metallic elements in these herbs is the main reason for a better quality of life for people (Ibourki et al. 2023; Jin and Arroo 2023; Madhab et al. 2023; Qadir et al. 2025). There are some misconceptions about the efficacy of traditional medicines. Since the beginning of human culture, plants have always been central to human survival and health. Humans early on realized the difference between plants that are fit for eating and plants that have medicinal properties. This knowledge gradually took shape and ultimately became traditional medicine in different cultures. At the present time, medicinal plants, herbal extracts, and their constituents are still in medical use, offering diverse biological functions, and are at the center of the research of this field (Pohl et al. 2016). The fact is that extensive scientific evidence supports their remarkable therapeutic value (Dalamagka 2024; Jansen et al. 2020); around 80% of people still depend on natural remedies as the first choice of healthcare (Sapoliya and Shah 2022).

Among the plants, ferns (Polypodiopsida), a group of vascular plants that reproduce via spores and lack seeds and flowers, are utilized as medicinal plants. The maidenhair fern *(Adiantum capillus‐veneris* L.) is a very popular medicinal herb with a tradition of use that goes back to times of yore and many tribes. The fern is used in the fight against several respiratory problems, such as cough, bronchitis, and asthma, as well as other applications, such as control of fever, treatment of skin diseases, normalization of the liver, and treatment of hair‐related diseases (hair‐loss), kidney stones, allergy, and hypertension (Naqishbandi 2014).

*Adiantum capillus‐veneris* stands out due to its wide geographical distribution. The reports on ethnobotanical and phytochemicals are available, yet no consolidated information summarizing their therapeutic relevance, phytochemistry, and potential biomedical applications exists (Jaafer et al. 2025). In addition, no comprehensive review has critically examined its medicinal value in connection with the current health challenges, such as antimicrobial resistance and chronic inflammatory conditions. This gap is our target by dealing with this underexplored species in the review, and we bring out the need for more pharmacological and clinical study of *Adiantum capillus‐veneris* L. Such an approach will provide instrumental support to future research work and lead to the creation of herbal therapies based on evidence. In addition, this review aims to show the historical relevance and modern use of medicinal plants in health care and to further support the emphasis on the therapeutic properties and the phytochemical composition of *Adiantum capillus‐veneris* L. There is also an emphasis on the necessity of examining the synergistic and antagonistic interactions of the natural compounds to make them function optimally for therapy.

## 2 The Taxonomy

Class: Pteridopsida; Order: Pteridales; Kingdom: Plantae; Division: Pteridophyta; Family: Adiantaceae, Genus: *Adiantum*, Species: *Adiantum capillus‐veneris* (Ahmed et al. 2012; Al‐Snafi 2015a; Kumar et al. 2023).

## 3 General Names

### 3.1 In Arabic Regions Name

Shaarul‐arz, Shaar‐ul‐jin, Kuzburat‐el bir, Shaar‐ul‐jibal, **for English**: Maiden hair fern, Our Lady's hair; Maria's fern, Gujarati: Hanspadi; in the language **Kurdish:** Bareze, Kizberet el‐bîr, Qeyteran, Xaleres, Xencerok, **Ayurvedic**: Hansaaraja, hansapadi; for language **Hindi**: Hansraj, Mubaraka, Pursha; **Kannada, Persia‐ushan; Unani, Tamil:** Hansraj, Sirsiapeshane, Seruppadai, Dumtuli, Barsioshan, Kazbaratul Ber (Al‐Snafi 2015a).

## 4 History and Botanical Description

People in different countries have applied the maidenhair fern ( *Adiantum capillus‐veneris* ) to different health complaints. In Iran, it was often used in a traditional method of medicine, known as “Pare‐siavashan,” mainly for lung complaints like asthma, cough, and chest pain, as well as for diuretic, anti‐inflammatory, and hair tonic purposes. In India, under Ayurvedic practices, it has been utilized to treat respiratory disorders, fever, and digestive issues. In ancient Greek and Roman medicine, Dioscorides and Galen documented its use for spleen pain, kidney stones, jaundice, and as a hair growth stimulant.

Furthermore, in Middle Eastern countries such as Iraq, it was a traditional practice to use the rhizome for coughing and pertussis. Furthermore, there is established evidence that in ancient Chinese medicine, *Adiantum capillus‐veneris* was used as a remedy for bronchitis, skin diseases, nephritis, and also served as a diuretic, among other applications. These varied applications are the very illustration of the broad therapeutic uses of this herb by different cultural traditions (Akbar and Akbar 2020; Dehdari and Hajimehdipoor 2018; Schwartz 2012). In Chinese medicine, the use of *Adiantum capillus‐veneris* has been a common practice since ancient times. The usual prescription includes the treatment of respiratory manifestations such as cough, fever, cold, and bronchial disorders, as well as conditions of the skin, liver, and tumors of the internal organs. Furthermore, especially in the southern areas of China, it was widely used in the past as a folk medicine for fighting inflammatory diseases, including gastritis, bronchitis, nephritis, dermatitis, and cystitis. The major bioactive compounds of the herb, such as flavonoids and phenolic acids, are responsible for its anti‐inflammatory, antimicrobial, and antioxidant properties.

**FIGURE 1:**
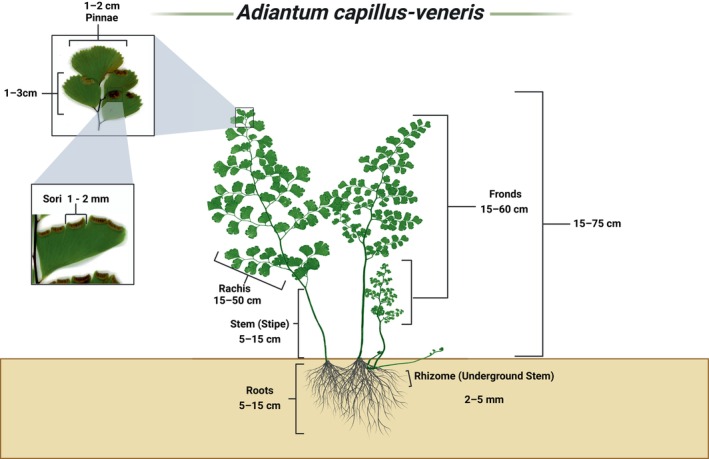
Morphological features and dimensions of *Adiantum capillus‐veneris* : This depiction points out the main elements of *Adiantum capillus‐veneris* : Mostly pinnate fronds (15–60 cm), rachis (15–50 cm), and stem (5–15 cm) as its parts. It also shows roots extending 5–15 cm deep and an old rhizome with a diameter ranging from 2–5 mm. The drawings that are given explain the sori for (1–2 mm) and pinnae for (1–3 cm long, 1–2 cm wide). Here you can see the complicated composition of such a fragile plant.

Moreover, up‐to‐date research has also shown the traditional use to be correct since the plant's extracts are seen to suppress the inflammatory mediators by inhibiting the activation pathway of NF‐κB. (Jiang et al. 2011; Rastogi et al. 2018; Yuan et al. 2012; Yuan et al. 2013). The *Adiantum capillus‐veneris* L. (Maidenhair fern) is a clump‐forming fern that is a member of the Pteridaceae (Dehdari and Hajimehdipoor 2018). A tiny, rhizome, intermediate, and a persistent living plant grows up to 35 cm in height with a black and wiry stipe. (Dehdari and Hajimehdipoor 2018; Rabiei and Setorki 2019). Figure 1 shows the morphological features of *Adiantum capillus‐veneris* as a delicate fern. It looks like a fan with sails, which are the pinnately compound fronds. They are usually about 15–60 cm in length, with individual leaflets (pinnae) that are between 1–3 cm long and 1–2 cm wide. They are bright green with a shiny, black rachis, which is up to 15–50 cm long. The stipe, a stem of 5–15 cm in length, joins the frond to the rhizome and has a slender and smooth body.

The rhizomes are creeping and scaly, about 2–5 mm in diameter, and can spread horizontally for a few centimeters. Pinnae are small, round to oblong, and alternately arranged along the rachis. Spore‐producing sori 1–2 mm in diameter can be found on the lower surface of the leaflets near the margins. In other words, they are also protected behind the reflexed edges. The fibrous roots, which are derived from the rhizome, are thin and hair‐like, measuring 1–3 mm in diameter. Usually, roots and rhizomes of this plant rise up to 5–15 cm from the soil surface to get moisture in the damp places it likes to grow, but some can grow 1.5–2 m. This fern can grow to a height of 15–75 cm and is mostly distributed in wet locations such as riverbanks and cliffs. (Al‐Snafi 2015b; Mickel 2004).

The plant is a thalophyte that requires moisture and shading at the same time; its usually linked to limestone rocks and calcareous covering a wide range of geographical regions, including the Americas, Europe, Africa, and Asia. Additionally, the plant is highly resilient to desiccation and exhibits adaptations such as specific leaf area (SLA) changes and efficient dark respiration under environmental stress, which support its survival in diverse ecological conditions (Anderson 2023). *Adiantum capillus‐veneris* is a kind of plant that is identified as a strong and solid species adapted to the changeable atmospheric conditions.

## 5 Chemical Properties of *Adiantum capillus‐veneris*

*Adiantum capillus‐veneris* is scientifically intricate, and at the same time, it is multifaceted with its health‐related capabilities. The crude mixture present in the plant and its diverse oil formulations includes essential oils, flavonoids, triterpenoids, phenylpropanoids, carotenoids, and minerals. Examination of the complex chemical data obtained by gas chromatography–mass spectrometry (GC–MS) and high‐performance liquid chromatography (HPLC) has paved the way for the elucidation of the plant's therapeutic properties. The essential oil of *Adiantum capillus‐veneris* is a complex of volatile products formed through the process of distillation (Khodaie et al. 2015). GC–MS is one of the most crucial and useful methods in chemical analysis. A total of 55 compounds detected have been recognized; the most important ones are carvone (33.00%), carvacrol (15.05%), hexadecanoic acid (7.02%), hexahydro‐farnesyl acetone (4.25%), and n‐nonanal (4.2%) (Talebi et al. 2022). Additionally, researchers found that carvone shows the highest (31.58%) concentration and that other compounds such as carvacrol (13.75%), hexadecanoic acid (5.88%), and thymol (4.05%) are also present (Khodaie et al. 2015). The essential oil, after hydrodistillation, has a yield of around 0.55% v/w. In addition, the oil has a peculiar smell and a very high level of antioxidant activity, and among its products' characteristics is the use of DPPH of 0.039 mg/mL to score its IC50 value (Khodaie et al. 2015). Terrestrial and extraterrestrial materials, such as carvone, carvacrol, and thymol, form the main basis of the free radicals' scavenging capacity, which is responsible for their antioxidative effect. In the experiment undertaken, the commercial oil product was obtained by the steam distillation of *Adiantum capillus‐veneris* (Khodaie et al. 2015).

**FIGURE 2:**
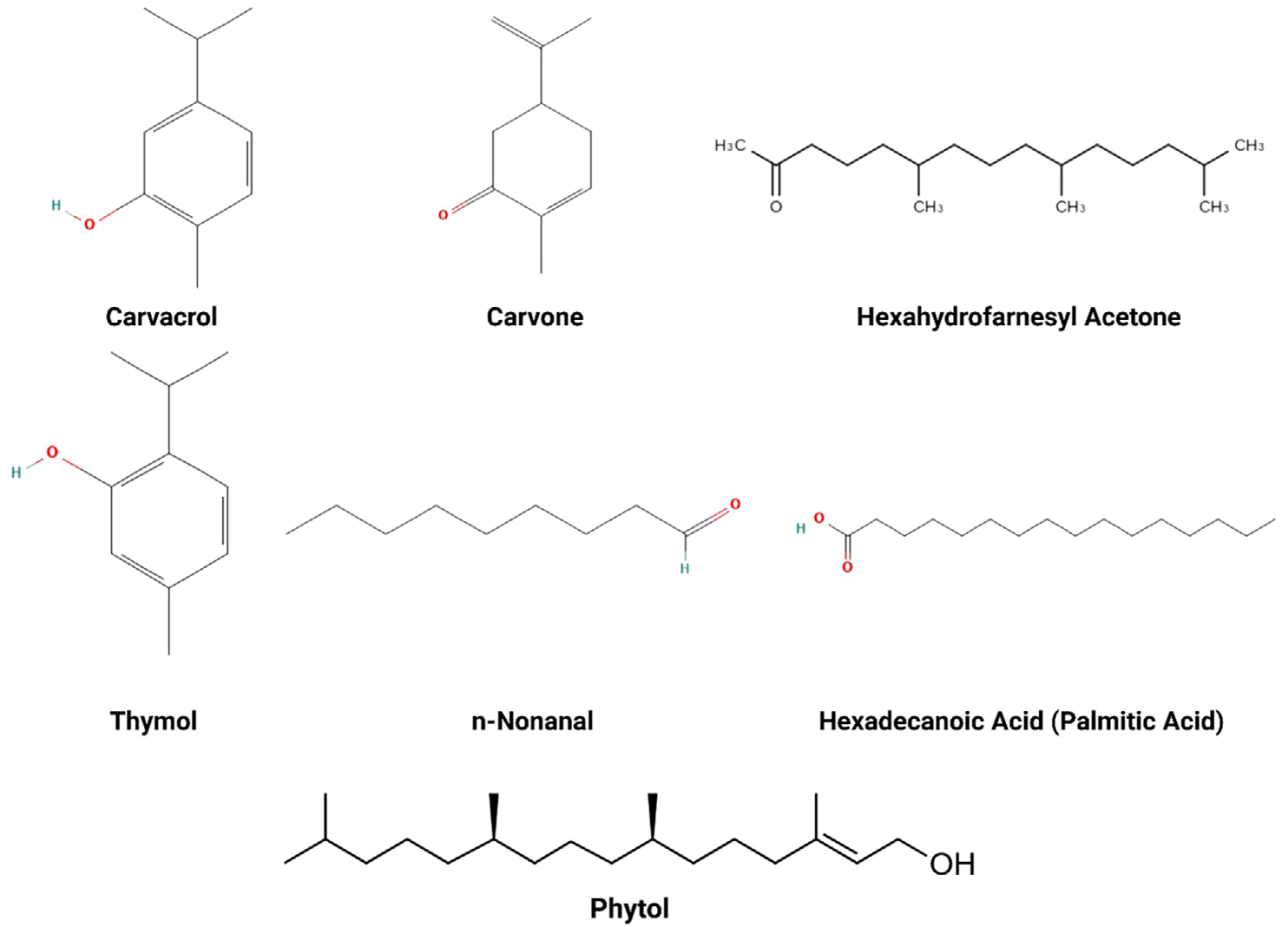
Chemical structures of major bioactive compounds identified in the essential oil of *Adiantum capillus‐veneris* . The depicted compounds include carvacrol, carvone, hexahydrofarnesyl acetone, thymol, *n*‐nonanal, hexadecanoic acid (palmitic acid), and phytol.

The compositional analysis of the essential oils was applied by the gas chromatography–mass spectrometry method, which is an approach that disclosed the presence of 12 components, including carvone and caryophyllene (Victor et al. 1970). The chemical composition of the essential oil extracted from *Adiantum capillus‐veneris* is shown in Table 1 with several major bioactive compounds, notably carvone, carvacrol, hexadecanoic acid (palmitic acid), hexahydrofarnesyl acetone, n‐nonanal, and thymol as illustrated in Figure 2. The presence of flavonoids already occupies a prominent position among the bioactive compounds in *Adiantum capillus‐veneris* . The chief ones of these belong to the flavonols and include quercetin, iso‐quercitrin, rutin, and astragalin (Akabori and Hasegawa 1969). The analysis of *Adiantum capillus‐veneris* has revealed a substantial total flavonoid content, which contributes significantly to its antioxidant and medicinal properties. To further characterize its phenolic profile, HPLC‐DAD‐MS analysis identified a wide range of phenolic acids and flavonoids (Table 2). Among the phenolic acids detected were gentisic acid (72.8 μg/g), chlorogenic acid (60.9 μg/g), p‐coumaric acid (19.9 μg/g), caffeic acid derivatives (51.8 μg/g), ferulic acid (183.8 μg/g), and 3‐p‐coumaroylquinic acid (325.8 μg/g). The flavonoid compounds included kaempferol (149.7 μg/g) and quercetin (108.1 μg/g), both recognized for their strong antioxidant potential. Together, these findings emphasize that the rich flavonoid and phenolic composition of *Adiantum capillus‐veneris* underpins its pharmacological activities and supports its traditional use as a medicinal plant. The aluminum chloride colorimetric method was carried out, and the result was that the flavonoid amount was 4.66 mg catechin equivalent per gram of dry weight (mg CE/g DW) (Boukada et al. 2022). The presence of chlorogenic acid, caftaric acid, and 5‐caffeoylquinic acid determines the superiority of the plant's antioxidant attributes. In addition, the antioxidant properties exhibited by these flavonoids, namely rutin and quercetin, are associated with their hypoglycemic and anti‐inflammatory activities. The aforementioned substances have the potential to attenuate oxidative stress and diabetes‐associated health problems (TBR) (Al‐Hallaq et al. 2015).

**TABLE 1:** The chemical compounds of *Adiantum caillus‐veneris* determined by GC‐Mass for essential oil compounds are listed along with their respective Kovats retention indices (KI) and percentage composition (HD%) (Nasrollahi et al. 2022).

Number	Compounds	KI	HD (%)	Number	Compounds	KI	HD (%)
1	4‐octen‐3one	956	0.14	29	Dodecanal	1384	0.19
2	1‐ octen‐ 3ol	961	0.08	30	N‐tetradecane	1403	0.70
3	N‐octanal	992	0.30	31	Alpha‐Ionone	1407	0.22
4	1,8‐cineol	1028	0.22	32	(Z)‐beta‐farnesene	1450	0.21
5	Alpha‐limonene	1032	0.04	33	Alpha‐santalene	1461	0.15
6	2‐octenal (E)	1034	0.14	34	Beta‐ionone	1462	0.92
7	2‐octen‐1‐ol	1045	0.16	35	Alpha‐curcumene	1473	0.31
8	1‐octanol	1060	0.25	36	E‐2‐tridecenal	1477	0.12
9	N‐nonanal	1082	4.20	37	Alpha‐murolene	1499	0.26
10	L‐linalool	1102	0.79	38	N‐pentadecane	1502	2.20
11	Camphor	1126	0.22	39	Calamenene	1515	0.21
12	Menthone	1152	0.32	40	Delta‐cadinene	1520	0.18
13	Borneol	1155	0.11	41	Endo‐1‐bourbonanol	1538	0.3
14	Nonanol	1156	0.17	42	Caryophyllenyl alcohol	1566	0.52
15	Terpineol‐4	1168	2.08	43	Cubenol	1623	0.10
16	Myrtenal	1168	0.19	44	Beta‐tumerone	1634	0.12
17	Cis—	1206	0.91	45	Cadalin	1656	0.21
	dihydrocarvone						
18	Cuminic aldehyde	1221	0.89	46	Jatamansone	1657	0.14
19	Carvone	1222	33.00	47	2‐pentadecanone	1674	0.10
20	Nerol	1226	0.14	48	Hexadecanal	1695	0.25
21	Trans‐anethol	1261	2.85	49	Heptadecane	1702	0.70
22	Thymol	1268	2.95	50	Octadecane	1803	0.05
23	Carvacrol	1278	15.05	51	Hexahydrofarnesyl acetone	1834	4.25
24	N‐undecanal	1282	0.12	52	Hexadecanoic acid	1919	7.02
25	2,4‐decadienal	1285	0.39	53	Phytol	2106	3.50
26	Alpha‐terpinyl acetate	1333	0.41	54	Linoleic acid	2087	0.89
27	Beta‐bourbonene	1380	0.30	55	Oleic acid	2096	0.91
28	Delta‐selinene	1384	0.50				

**TABLE 2:** Phenolic compounds identified in *Adiantum caillus‐veneris* using HPLC‐DAD‐MS (Boukada et al. 2022).

Compounds	Rt (min)	Extract (μg/g)
Gentisic acid	3.52	72.8
Chlorogenic acid	6.43	60.9
p‐coumaric acid	9.92	19.9
Caffeic acid derivatives	11.07	51.8
Ferulic acid	12.14	183.8
Kaempferol	15.49	149.7
Quercetin	25.73	108.1
3‐p‐Coumaroylquinic acid	29.54	325.8
Apigenin‐7‐O‐glucoside	33.97	353.4
3,5‐Di‐O‐caffeoylquinic acid	36.03	769.0
Isorhamnetin‐3‐O‐di‐glucoside	37.37	257.3
Quercetin‐3‐O‐glucoside	41.21	949.5
Epicatechin‐7‐O‐rutinoside	42.15	255.5
Kaempferol‐3‐O‐glucoside	45.62	359.6
Ferulic acid derivatives	46.46	110.1
Hydroxycinnamic derivative	48.47	404.3
Ferulic acid derivatives	50.30	219.6

*Note:* Extract (μg/g): Concentration of compound in the hydromethanolic extract of *Adiantum capillus‐veneris* leaves.

Abbreviation: Rt (min), Retention time in minutes.

Flavonoids occupy a prominent position among the bioactive compounds in *Adiantum capillus‐veneris* . Key examples include astragalin, quercitrin, naringin, populin (nicotiflorin), procyanidin, and prodelphinidin, which have been reported in its fronds (Vadi et al. 2017). In a separate column, HPLC‐DAD has been employed to quantify carotenoids; for example, lutein (806.0 μg/g), chlorophyll b' (410.0 μg/g), and kaempferol‐3‐sophorotrioside (58.7 mg/g), along with one of the phenolic acids, chlorogenic acid (28.5 mg/g), are the main actors (Zeb and Ullah 2017). Additionally, diazoxide, a triterpenoid epoxide derived from filic‐3‐ene, has been characterized through NMR and mass spectrometry. Such compounds are formed from squalene via extensive rearrangements, highlighting the complex biosynthetic pathways of the plant (Ibraheim et al. 2011). Saponins, another key class of phytochemicals, provide surface‐active properties and aid in reducing cholesterol levels. Together with triterpenoids, these compounds contribute to the plant's anti‐inflammatory, antibacterial, and antifungal activities (Moghimipour and Handali 2015). On the other hand, the mineral analysis of *Adiantum capillus‐veneris* , as presented in Table 3, revealed the presence of 10 essential elements in varying concentrations, determined by ICP‐AES. The most abundant elements were potassium (17.95 mg/g) and calcium (11.52 mg/g), followed by magnesium (2.90 mg/g) and iron (17.45 mg/100 g). Other elements detected in smaller amounts included manganese (13.55 mg/100 g), sodium (12.65 mg/100 g), zinc (7.15 mg/100 g), copper (1.70 mg/100 g), and nickel (0.20 mg/100 g), while cobalt was not detected. Overall, the descending order of elemental concentration was K > Ca > Mg > Fe > Mn > Na > Zn > Cu > Ni. This mineral profile highlights the nutritional and therapeutic potential of *Adiantum capillus‐veneris* , particularly due to its high potassium and calcium levels, which are essential for physiological processes.

**TABLE 3:** Elemental concentrations in *Adiantum capillus‐veneris* foliar tissue (determined by ICP‐AES) (Rajurkar and Gaikwad 2012).

Elements	Concentration (mg/g)	Concentration (mg/100 g)
Calcium (Ca)	11.52	—
Potassium (K)	17.95	—
Magnesium (Mg)	2.90	—
Iron (Fe)	—	17.45
Manganese (Mn)	—	13.55
Sodium (Na)	—	12.65
Zinc (Zn)	—	7.15
Copper (Cu)	—	1.70
Nickel (Ni)	—	0.20
Cobalt (Co)	—	N.D.

## 6 Methods of Compounds Extraction From *Adiantum capillus‐veneris*

### 6.1 Water Extraction

**FIGURE 3:**
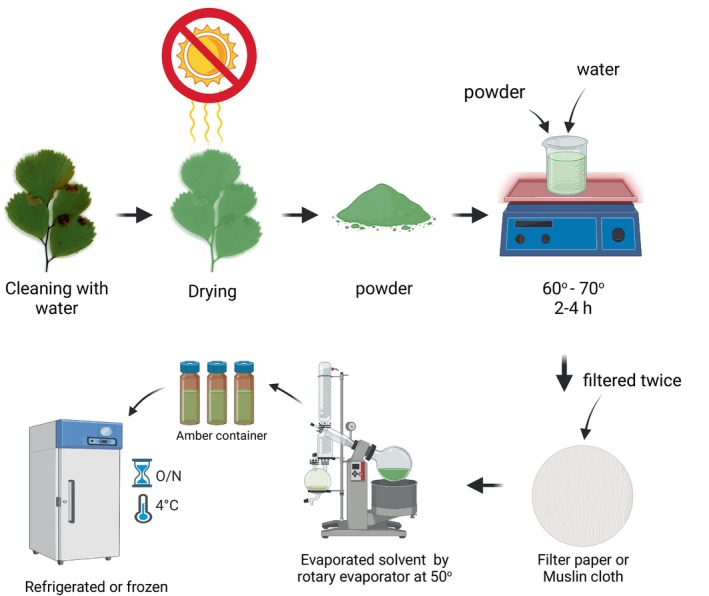
Methods of water extraction.

Water extraction of *Adiantum capillus‐veneris* bioactive substances is common. This approach extracts hydrophilic chemicals such as phenolics, flavonoids, tannins, and others using water. First, leaves are cleaned, air‐dried in the shade to retain delicate chemicals, and then milled into fine powder. To extract efficiently, this powdered material is combined with distilled water in a conical flask and heated at 40°C for 2–4 h while stirring. The heated mixture is cooled and filtered using fine filter paper or muslin cloth to separate the liquid extract from the plant detritus. The filtration phase is repeated twice with fresh water to enhance bioactive chemical output. Then concentrate the mixed filtrates by evaporating water under reduced pressure or gently heating in a water bath and keeping the temperature below 40°C to preserve compound bioactivity. To prevent degradation, the concentrated aqueous extract is refrigerated or frozen in a sterile, amber container (Chemat and Strube 2015; Rajurkar and Gaikwad 2012). Figure 3 summarizes the step‐by‐step extraction compound used for water. Table 4 shows the extraction methods and compounds extracted from *Adiantum caillus‐veneris*.

**TABLE 4:** Extraction methods and compounds extracted from *Adiantum caillus‐veneris*.

Extraction method	Compounds extracted
Microwave‐assisted extraction (MAE)	Phenolic acids (chlorogenic acid, caffeic acid), flavonoids (kaempferol, quercetin derivatives), carotenoids (lutein), saponins
Supercritical fluid extraction (SFE)	Essential oils (carvone, carvacrol, thymol), fatty acids (palmitic acid, hexadecanoic acid), terpenoids
Soxhlet extraction	Polyphenols (flavonoids, tannins), fatty acids (palmitic acid, linoleic acid), phenolic acids (ferulic acid)
Cold maceration	Phenolic acids (chlorogenic acid, p‐coumaric acid), flavonoids (kaempferol glycosides, quercetin glycosides), tannins
Ultrasound‐assisted extraction (UAE)	Saponins, phenolics (caffeic acid, ferulic acid), flavonoids (quercetin, kaempferol‐3‐O‐glucoside)
Hydrodistillation	Essential oils (carvone, carvacrol, thymol, nonanal, hexadecanoic acid), volatile terpenoids
The reversed‐phase high‐performance liquid chromatography	Carotenoids (lutein, violaxanthin), chlorophylls (chlorophyll a, chlorophyll b'), phenolic acids (chlorogenic acid, 5‐caffeoylquinic acid), flavonoids (kaempferol glycosides, quercetin glycosides)

### 6.2 Reversed‐Phase HPLC for Chlorophylls and Phenolic Extraction

**FIGURE 4:**
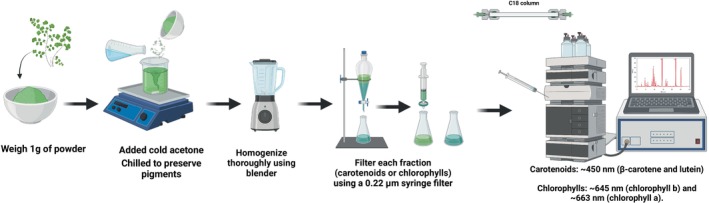
Method of reversed‐phase HPLC extraction.

RP‐HPLC is a precise method for separating, identifying, and quantifying bioactive components from *Adiantum capillus‐veneris* . The method chromatographically separates plant extracts using a nonpolar stationary phase (e.g., C18 column) and polar mobile phases for specific chemical classes. The hydrophobic interactions between molecules and the stationary phase enable effective separation. Additionally, RP‐HPLC is ideal for identifying plant extract phenolic acids, flavonoids, carotenoids, and chlorophylls shown in Figure 4. Carotenoids and chlorophylls are also separated by using a tertiary gradient system of water, MTBE, and methanol–water in *Adiantum capillus‐veneris* study. A binary gradient method of methanol–water‐acetic acid optimizes hydrophilic and hydrophobic phenolic component resolution for phenolic profiling (Zeb and Ullah 2017). A diode array detector (DAD) improves detection sensitivity and allows simultaneous monitoring of substances at their UV–Vis absorption wavelengths.

### 6.3 Hydro‐Distillation

**FIGURE 5:**
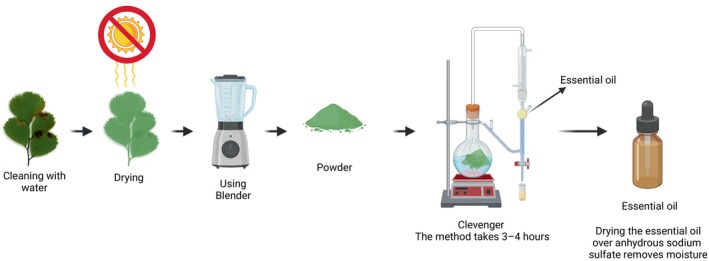
Method of hydro‐distillation.

Hydro‐distillation is frequently used to extract volatile chemicals and essential oils from plants like *Adiantum capillus‐veneris* . The method immerses plant material in water or above boiling water to allow steam to travel through its tissues. Thus, the steam carries the essential oil into a cooling system after heating and steaming break the volatile oil cells. Then the oil is separated from the water due to its immiscibility and density after collecting the condensate. In hydro‐distilling, either fresh or dried aerial components of *Adiantum capillus‐veneris* are introduced into the process (Pheko‐Ofitlhile and Makhzoum 2024). A Clevenger device is commonly used for this process. For efficient volatile component extraction, the method takes 3–4 h, as shown in Figure 5. Drying the essential oil over anhydrous sodium sulfate removes moisture and preserves its chemical integrity in amber bottles at low temperatures.

### 6.4 Ultrasound‐Assisted Extraction (UAE)

**FIGURE 6:**
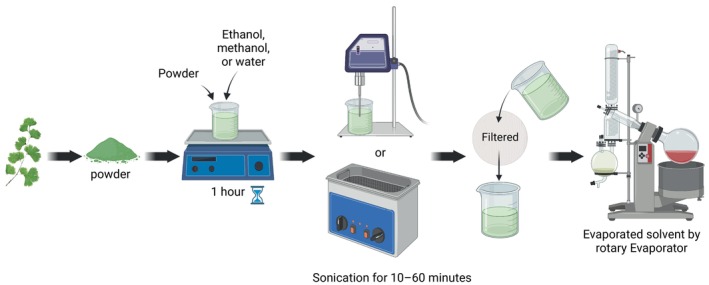
Method of ultrasound‐assisted extraction (UAE).

The contemporary and effective ultrasound‐assisted extraction (UAE) method in isolation of bioactive components from *Adiantum capillus‐veneris* . Ultrasonic waves cause solvent cavitation bubbles that collapse and generate localized high pressure and temperature, shown in Figure 6. The disruption of plant cell walls increases intracellular chemical release into the solvent. The UAE is useful for extracting delicate chemicals at low temperatures without affecting bioactivity. The UAE is also used as a controlled ultrasonic bath or probe system to mix dried and powdered plant material with ethanol, methanol, or water (Shen et al. 2023). Depending on the chemical composition of the sample and optimization parameters, including ultrasonic frequency, amplitude, and solvent type, sonication typically takes 10–60 min. Following sonication, the extract is filtered, concentrated, and subjected to further analysis.

### 6.5 Microwave‐Assisted Extraction (MAE)

**FIGURE 7:**
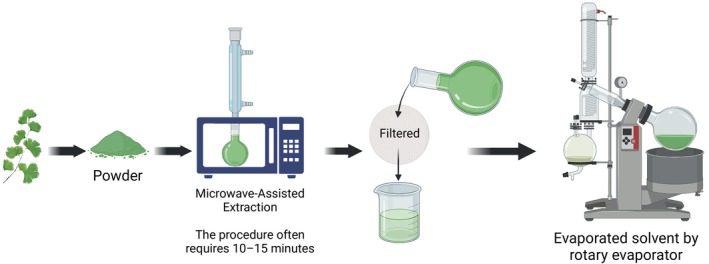
Method of microwave‐assisted extraction.

Extracting bioactive compounds from *Adiantum capillus‐veneris* is a technique called microwave‐assisted extraction (MAE) technology, which is a method based on the principle of using microwaves to heat the solvent and plant material very fast, disrupting cell walls, and thus enhancing the release of intracellular compounds (López‐Salazar et al. 2023). Microwave power, the length of time, and the amount of solvent are the parameters that need to be adjusted when the greatest yield is sought. The process is frequently only 10–15 min long, which is represented in Figure 7 and sharply cuts the duration in comparison with the conventional techniques.

### 6.6 Supercritical Fluid Extraction (SFE)

**FIGURE 8:**
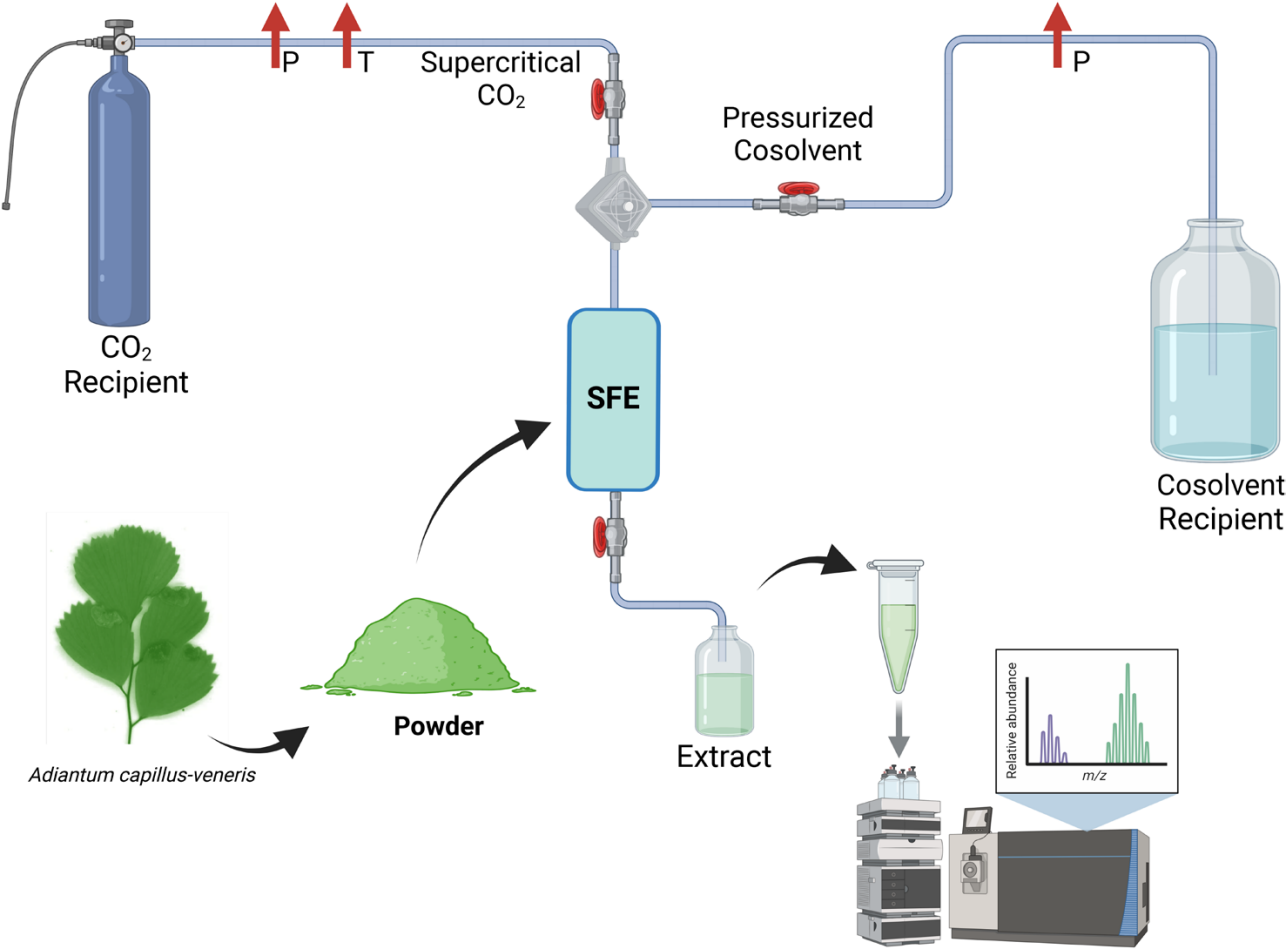
Method of supercritical fluid extraction.

Supercritical fluid extraction (SFE) is a very effective and precise technique that is commonly used for bioactive compounds to be extracted from *Adiantum capillus‐veneris* . The procedure applies a supercritical form of CO_2_, and most commonly, co‐solvents like ethanol or methanol are used in the process as the extracting fluid. Under the supercritical condition, CO_2_ is known to have properties that are quite similar to both gas and liquid; the low viscosity, high diffusivity, and very strong solvating power make it the best choice for separating nonpolar and moderately polar compounds (Herzyk et al. 2024). This method is well‐known for its eco‐friendly approach, demonstrated in Figure 8, as it does not require toxic solvents and minimizes thermal degradation of sensitive compounds.

An evaluation on the performance, cost, and scalability of each method is reviewed in Table 5 to give practical guidance for understanding its extraction applicability, allowing industry professionals to choose the most relevant technique for desired compound isolation with what known resources and at what scale application from the industry perspective. Microwave‐assisted extraction (MAE) and ultrasound‐assisted extraction (UAE)—both with high efficiency for semi‐industrial level production, supercritical fluid extraction (SFE)—for high‐value compounds, although equipment cost may apply. In contrast, solvent extraction and hydrodistillation are more hydraulically accessible, but they exhibit low efficiency and relatively modest selectivity. Scalability aside, the extracted compounds still need to be analyzed by reversed‐phase HPLC for analytical profiling and quality assurance. Such comparative values could improve the translation of laboratory research with *Adiantum capillus‐veneris* to commercial applications.

**TABLE 5:** Comparative evaluation of extraction methods for *Adiantum capillus‐veneris*.

Extraction Method	Efficiency	Cost	Scalability	Advantages	Limitations	References
Solvent extraction	Moderate	Low	High	Simple, low‐tech, traditional	Long extraction time, solvent disposal issue, and low selectivity	[41, 42]
Ultrasound‐assisted extraction (UAE)	High	Moderate	Moderate	Short time, low temp, eco‐friendly	Equipment cost, scaling limitations	[45]
Microwave‐assisted extraction (MAE)	Very High	Moderate‐High	Moderate	Fast, high yield, low solvent use	May degrade heat‐sensitive compounds	[46]
Supercritical fluid extraction (SFE)	Very High	High	Low‐Moderate	Green, solvent‐free, selective	Expensive equipment, technical skill needed	[47]
Hydrodistillation	Low‐Moderate	Low	Low	Traditional, simple equipment	Heat‐sensitive compound degradation, energy‐intensive	[44]
Soxhlet Extraction	Moderate‐High	Low‐Moderate	Low	Exhaustive extraction	Long time, large solvent volume, thermal degradation	[10]
Cold maceration	Low	Very Low	Low	Minimal equipment preserves heat‐sensitive compounds	Low yield, long duration	[10]
Reversed‐phase HPLC (RP‐HPLC)	Very High (analytical precision)	High	Not scalable	Excellent profiling and compound purity	Not suitable for mass extraction	[43, 38]

## 7 Applications and Functional Characteristics of *Adiantum capillus‐veneris*

**FIGURE 9:**
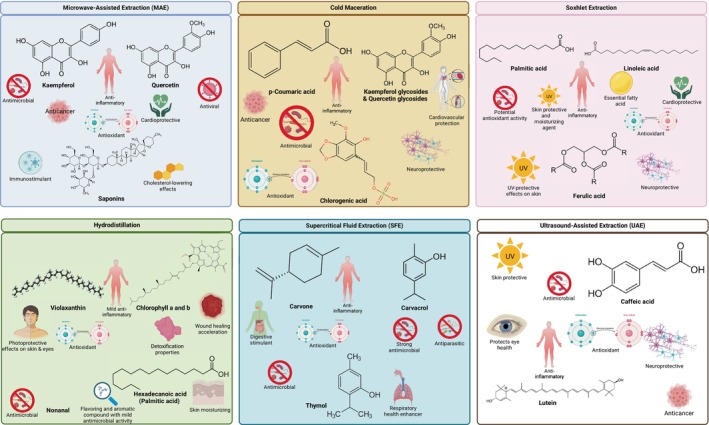
Bioactive compounds from *Adiantum capillus‐veneris* extracted by MAE, UAE, SFE, Soxhlet, cold maceration, and hydrodistillation. These methods yield flavonoids, phenolic acids, and essential oils with antimicrobial, antioxidant, anti‐inflammatory, neuroprotective, and cardioprotective activities.

Recent advancements in extraction technologies have allowed for the targeted isolation of bioactive compounds from *Adiantum capillus‐veneris* , each method favoring specific classes of phytochemicals with distinct pharmacological properties. As illustrated in Figure 9, modern techniques such as microwave‐assisted extraction (MAE), ultrasound‐assisted extraction (UAE), and supercritical fluid extraction (SFE) are highly effective in recovering potent antioxidant, antimicrobial, anti‐inflammatory, and neuroprotective agents, including kaempferol, quercetin, carvone, carvacrol, caffeic acid, and chlorogenic acid. These compounds exhibit various therapeutic actions—ranging from antioxidant defense and skin protection to cardiovascular support and antimicrobial effects. By integrating advanced green extraction techniques, researchers can enhance the yield and bioefficacy of these constituents while minimizing degradation, paving the way for potential pharmaceutical, nutraceutical, and cosmetic applications of *Adiantum capillus‐veneris* .

### 7.1 Maidenhair Fern Traditional Uses in Herbal Formulations

*Adiantum capillus‐veneris* is a prevalent species with significant potential for medicinal and nutritional applications. Species of *Adiantum* were utilized for thoracic ailments, coughs, and respiratory infections; as an expectorant; to enhance breastfeeding; to support renal function; for antiparasitic purposes; and for dandruff treatment. The medicinal properties of the fresh or dried leafy fronds include acting as an antidandruff, antitussive, astringent, demulcent, depurative, emetic, galactagogue, mild emmenagogue, emollient, moderate expectorant, febrifuge, laxative, pectoral, refrigerant, stimulant, sudorific, and tonic. A similar purpose was served by the tea made from the dried plant fronds (Dehdari and Hajimehdipoor 2018; Rastogi et al. 2018; Vadi et al. 2017). The main multi‐herbal formulations of the *Adiantum capillus‐veneris* , along with their indications and dosage forms, are now in the markets in Table 6.

**TABLE 6:** Overview of market‐available products featuring *Adiantum capillus‐veneris* (maidenhair fern): Manufacturers, items, and key features.

The Company manufacturer	The Item	Features of the Product
Luxéol	Hair loss food supplements Luxeol 60 gummies	Montpellier capillary aerial portion dry extract
Mulato	Food supplements hair loss capsules M. expert Patrice Mulato	Montpellier Capillary (*Adiantum capillus veneris*)
Hawaii Pharm	Glycerite Herbal Supplement 2 × 32 oz	Maidenhair Fern (*Adiantum Capillus Veneris*) Glycerite, Organic Dried Herb Alcohol‐Free Liquid Extract
Herbal‐Terra	Promotes respiratory health and hair	Maidenhair Fern (*Adiantum Capillus Veneris*) Tincture Dried Herb Liquid Extract
Rainforest Pharmacy	Avenca Capsules ( *Adiantum Capillus‐veneris* ) 500 mg, 100 Capsules | Wildcrafted Maidenhair Fern Herbal Supplement	Each capsule contains 500 mg of wildcrafted Avenca leaves and rhizomes
bixa BOTANICAL	bixa BOTANICAL Ayurvedic Natural Herbal Supplement Maidenhair Fern Powder Pure Authentic Premium Quality ‐ 7 Oz (200 g) (*Adiantum Capillus*/Hansraj), Supports Immunity	Maidenhair Fern Powder Pure Authentic Premium Quality
Terravita	Maidenhair Fern (450 mg, 100 Capsules)	Maidenhair Fern—450 mg
BIOCYTE LABORATOIRE	Kératine Gummies 100 mg	Madenhair Fern is the plant par excellence that prevents hair loss. Each gummy contains 150 mg of Madenhair Fern.
Linea Mamma Baby	Doccia shampoo and shower gel	Mom and Dad Shower Shampoo 500 mL (cardamom, olive, maidenhair)
Prime HAIR CARE	Prime S+ Maidenhair Sebum Control Shampoo	Madenhair Fern extract

### 7.2 Antimicrobial Properties

*Adiantum capillus‐veneris* exhibits significant antimicrobial properties, attributed to its diverse phytochemical composition. Studies have demonstrated that extracts prepared using solvents such as water, methanol, and ethanol are effective against a variety of multidrug‐resistant (MDR) bacterial and fungal strains. The plant's antimicrobial efficacy is linked to its rich content of flavonoids, alkaloids, tannins, and other bioactive compounds, as shown in its ability to inhibit pathogens like *Staphylococcus aureus* , *Escherichia coli* , and *Klebsiella pneumoniae* through standard susceptibility tests such as disc diffusion and MIC evaluations (Ishaq et al. 2014).

Moreover, there has been a report that methanolic extracts from the fern prove to show remarkable growth inhibitory and bactericidal effects on clinically causing pathogens of both gram‐positive and gram‐negative profiles, as well as express antifungal activities toward clinical isolates (Hadi and Hussein 2016). In addition, the antibacterial efficacy of *Adiantum capillus‐veneris* has been extensively demonstrated against specific multidrug‐resistant (MDR) bacterial and fungal strains. Extracts derived from its leaf, stems, and roots have been tested against 10 MDR bacterial strains, including gram‐positive bacteria such as *Staphylococcus aureus* and gram‐negative bacteria such as *E. coli* , *Citrobacter freundii* , *Providencia*, *Pseudomonas aeruginosa* , *Klebsiella pneumoniae* , *Salmonella enterica serovar Typhi*, *Shigella*, *Proteus vulgaris* , and *Vibrio cholerae* . Among the tested solvents, methanol, ethanol, and water extracts showed stronger antibacterial activity compared to hexane extracts. The methanolic extract of the leaf exhibited the most substantial zones of inhibition against *Providencia*, *K. pneumoniae* , *Shigella*, *V. cholerae* , *S. aureus* , *P. vulgaris* , and *S. typhi* .

On the other hand, the methanolic extract obtained from the stems demonstrated the highest antibacterial activity against *E. coli* , *K. pneumoniae* , and *S. typhi* . Additionally, the aqueous extract of the leaf displayed broad‐spectrum antibacterial activity, effectively inhibiting all 10 bacterial strains. The stem aqueous extract also showed notable efficacy, particularly against *E. coli* , *K. pneumoniae* , *S. typhi* , *Shigella*, *P. vulgaris* , and *Providencia* (Ishaq et al. 2014).

Additionally, several preclinical studies demonstrate that the methanolic extracts of *Adiantum capillus‐veneris* aerial parts exhibit antimicrobial properties at concentrations ranging from 0.5 to 2 mg/mL against *Bacillus* sp., *E. coli, Staphylococcus* sp., *Proteus*, and *P. aeruginosa* (Hadi and Hussein 2016). At the same time, it was reported in another study that the methanol extract showed the lowest minimum inhibitory concentration (MIC) values, such as 0.48 μg/mL against the bacteria *E. coli* , and it was effective against G+ and G‐ bacteria and even multiresistant *S. aureus* and eight fungal strains, respectively (Singh et al. 2008).

Comparative evaluation of the antifungal effectiveness of the crude extracts and phenolic fractions of the plant parts and the gametophytes of *Adiantum capillus‐veneris* against *A. niger* and *Rhizopus stolonifer* was carried out. The results obtained as a result of the tests indicated that gametophytes have more prominent antifungal properties compared to the sporophyte parts, which then suggests that they may indeed be very effective in treating fungal diseases (Ghosh and Gupta 2005). It was also confirmed that the 100% ethanol extract of the rhizomes of *Adiantum capillus‐veneris* showed antiviral effects against vesicular stomatitis virus in vitro (Kashkooe et al. 2020; Rastogi et al. 2018). Clinical trials have illustrated that *Adiantum capillus‐veneris* is an effective broad‐spectrum antimicrobial agent. Its use for treating mouth ulcers, wounds, and respiratory tract infections has been traditional. Flavonoids and phenolic compounds found in plants have been shown to inhibit bacterial growth. In particular, it is mentioned in the ethnomedicinal records of India and Iran as a popular remedy for bacterial illnesses such as coughs and urinary tract infections. An extract obtained from the whole plant has been proven to be a great way of effectively combating oral infections as well as fungal infections (Jeyalatchagan Sureshkumar et al. 2018; Kimta et al. 2025).

### 7.3 Antioxidant Properties

Oxidative stress (OS) is a condition that occurs when there is an imbalance between prooxidants and antioxidants, where there are more prooxidants, leading to cellular damage. Reactive oxygen species (ROS) are needed for normal cellular functions that are well controlled by complicated mechanisms of redox regulation, receptor signaling, and the second messengers of chemical signaling (Schieber and Chandel 2014; Vardar Acar and Özgül 2023). Nonetheless, under pathological situations, the uncontrolled rise of ROS leads to malfunctions in the systems, causing oxidative stress, which subsequently results in damage and alterations to the signaling pathways of the cells (Ray et al. 2012). Recent research has improved our understanding of oxidative stress, showing how it disrupts thiol redox circuits and causes damage to large molecules. These disruptions can impair redox regulation and cellular signaling, significantly affecting physiological and pathological processes (Jones 2006, 2008). Also, the disruption of redox circuits by certain reactions involving these redox‐sensitive components alters electron transfer or breaks the gating mechanism, which is vital in both physiopathology and physiology (Ray et al. 2012).

Oxidative stress is implicated in the oxidation of proteins, disruption of cellular signaling, and impairment of critical biological functions, including vascular health, immune activation, and cardiovascular remodeling (Yung et al. 2006). It contributes to processes like inflammation, apoptosis, fibrosis, and cellular proliferation. ROS levels are elevated due to environmental toxins, pollutants, and viral infections, particularly in the respiratory system (Khomich et al. 2018). In this way, the infections are also engaged to produce this excessive ROS and, further, lower the antioxidant defenses, which in turn increases oxidative damage and promotes the disease progression. Cellular functions are affected by ROS by means of adding posttranslational modifications, for instance, by the way of phosphorylation, nitrosylation, or sulfenylation. It has been noted that proteins such as receptors, ion transporters, transcription factors, and structural molecules are especially prone to suffer from dysregulation in the vascular, cardiac, and renal functions (Foo et al. 2022; Garofalo et al. 2013; Khomich et al. 2018; Komaravelli and Casola 2014). Proteins susceptible to the oxidative effects of reactive oxygen species (ROS) include receptors, ion transporters, signaling molecules, transcription factors, matrix metalloproteinases (MMP), and cytoskeletal structural proteins, all of which are involved in the regulation of vascular, cardiac, and renal functions; under normal circumstances, the nuclear factor erythroid 2‐related factor 2 (Nrf2) activates antioxidant defenses. Interestingly, it is possible to detect suppressed signals of Nrf2 activity during respiratory infections, which essentially reflects nuclear factor kappa‐light‐chain enhancer of B cells (NF‐kB) dominating the reaction caused by oxidative stress and cellular death (Santos et al. 2016; Xu et al. 2017).

Oxidative stress can also cause cell proliferation, death, and growth; frequently, the free radicals that harm the lipids and proteins, as well as the DNA, are the mediators. Reactive oxygen species such as superoxide ions and hydrogen peroxide, generated during cellular metabolism, cause significant oxidative harm to tissues through covalent bonding and lipid peroxidation. The determined treatment of health problems such as cancer, neurodegenerative diseases, and inflammation caused by oxidative stress relies on polyphenol‐rich foods as antioxidants, providing preventive and posttreatment support through neutralizing ROS (Daskou et al. 2023; Fratta Pasini et al. 2021). In addition, cell proliferation is stimulated by oxidative stress, apoptosis, inflammation, and hypertrophy by the activation of the redox chain processes. Free radicals have the potential to repeatedly lead to oxidative alterations of different compounds through the connecting pathways with other ROS.

Moreover, the oxidative damage of proteins and lipids brings about the injuries of VSMCs, endothelial cells, and cardiac cells, thus resulting in substantial adjustments of redox gene expression, increased intracellular calcium, and long DNA breaks occurring (Juan et al. 2021; Satoh et al. 2010). Reactive oxygen species, including singlet oxygen, superoxide ions, and hydrogen peroxide, are highly reactive and detrimental compounds produced by cells during metabolic processes. They induce considerable oxidative harm to proteins, lipids, enzymes, and DNA by covalent bonding and lipid peroxidation, leading to tissue injury (Benabderrahim et al. 2019). Natural antioxidants are highly regarded for their capacity to neutralize free radicals. Free radicals are linked to the onset of various diseases, including cancer, neurodegeneration, and inflammation (Baba and Malik 2015; Rakesh et al. 2010). Plant‐derived antioxidants, such as phenolics and flavonoids, may have preventive effects against several diseases (Gülcin 2012). Natural antioxidants, particularly plant‐derived compounds like phenolics and flavonoids, are increasingly valued for their role in combating oxidative damage. Among such plants, *Adiantum capillus‐veneris* has shown exceptional antioxidant potential. Studies from Algeria demonstrated that its hydromethanolic extract effectively scavenged DPPH radicals, with an IC_50_ value of 65.85 μg/mL, primarily attributed to quercetin 3‐O‐glucoside (Boukada et al. 2022). In Bangladesh, another research was conducted to study the methanolic extract and its fractions, which revealed that the ethyl acetate fraction had the highest antioxidant activity and IC_50_ value of 1.05 μg/mL in DPPH assays. Actually, the ratio was much better than the reference standard ascorbic acid, underscoring the remarkable free radical scavenging capacity of the plant (Roy et al. 2019). The leaves of *Adiantum capillus‐veneris* are the source for the ethanol extract that displays a remarkable ability to exert antioxidative action. The IC50 value of the sample in the DPPH assay was found to be as low as 0.3986 mg/g. These robust performances are almost certainly the consequence of the high levels of polyphenols and flavonoids in the plant (Rajurkar and Gaikwad 2012). The clinical trials with plant‐based flavonoids have shown that these stimulate antioxidant enzymes, including superoxide dismutase (SOD), catalase (CAT), and glutathione (GSH), while downregulating the markers of oxidative stress, such as malondialdehyde (MDA), in vertebrates (Jiang et al. 2011).

Furthermore, *Adiantum capillus‐veneris* hydroalcoholic extract was tested to be effective in reducing oxidative stress markers and increasing the overall antioxidant capacity in stress‐affected organisms. The fact that the plant can withstand oxidative damage demonstrates the possibility of using it as a source of natural antioxidants (Ahmadpoor et al. 2019; Ahmadpouri et al. 2020). The plant's methanolic extracts are a very good demonstration of the updated data about polyphenols, vitamin E, and antioxidant activity, which are related to the removal of free radicals (Ham et al. 2015; Roy et al. 2013). With its polyphenolic content, flavonoids, and phenolic acids, the plant demonstrates strong potential for neutralizing free radicals. Those substances work by processes that include hydrogen ion transfer and metal ion chelation. It has been mentioned in several studies that methanolic extracts of the ferns of *Adiantum capillus‐veneris* showed antioxidant properties, such as those that are attributed to its protective effects during the course of the birth of oxidative stress‐related disorders, including skin and inflammatory conditions (Farràs et al. 2022; Kimta et al. 2025).

In summary, *Adiantum capillus‐veneris* has incredible capabilities as a plant with strong antioxidant properties. The major components of this plant are phenolic and flavonoid compounds, of which the most important one is the quercetin derivative. The plant also contains antioxidants that can protect the human body from several diseases, like osteoarthritis, aging, and other neurodegenerative diseases. It can also be used to improve health, nutrition, and food preservation.

### 7.4 Anti‐Inflammatory Properties

Inflammation is a great and important job of the immune system, and it is only activated in certain circumstances, such as infections, tissue injuries, and toxic substances that can be harmful. The immune system is activated by a network of complex cytokines and their receptors, which are the ones that are responsible for the initial point of the inflammation. The main activity of the immune system in the fight against pollution is found in the recognition of the collection of danger molecules (DAMPs) and the development of cytokines, chemokines, and other pro‐inflammatory mediators. The inflammation response derives its significance from the fact that it seeks not only to get rid of the flammable agents but also to trigger the healing process and the restoration of the health system (Caballero‐Sánchez et al. 2024).

Furthermore, persistent systemic inflammation, powered by autoimmune reactions, together with oxidative stress causing slow and toxic cell damage, could be the main cause of some chronic diseases such as heart disease (Ahmed 2011; Megha et al. 2021; Yadav and Dange 2020). Inflammation is caused by layered mechanisms such as NF‐κB, MAPK, and JAK–STAT pathways that ensure that the genes related to immunity signaling and tissue regeneration are activated and expressed (Chen et al. 2015). Grasping the biological mechanisms of inflammation presents various options for therapies focused on certain goals, especially for the treatment of chronic inflammatory disorders (Libby 2007; Murakami and Hirano 2012). One important point when treating inflammation is the ability to dull the persistent inflammation‐generated damage while enabling the acute inflammatory process to resume naturally.

The most commonly used NSAIDs, for example, aspirin, ibuprofen, also called nonsteroidal medications, and key drugs such as corticosteroids work by inhibiting inhibitors of the pro‐inflammatory process like prostaglandins and cytokines, so the patient's pain and swelling can be abated. Monoclonal antibodies that target inflammation‐causing cytokines like TNF‐α and IL‐6 were brought in through biologic therapy and are now very common in the management of rheumatoid arthritis and Crohn's disease (Brenner and Krakauer 2003; Libby 2007). Emerging therapies include drugs that mimic pro‐resolving mediators, which actively promote the resolution of inflammation and tissue repair without suppressing the immune response (Dalli and Serhan 2018). Lifestyle interventions, such as anti‐inflammatory diets and exercise, complement medical treatments by addressing underlying systemic inflammation, particularly in metabolic and cardiovascular disorders (Ahmed 2011).

Presently, scientific researches uncover *Adiantum capillus‐veneris* as a drug with appreciable anti‐inflammatory effect. Specifically, ethanolic extracted contents of the plant have demonstrated a reduction in the levels of the inflammatory mediators, for instance, the prostaglandin E2 (PGE2), the interleukin 6 (IL‐6), and the tumor necrosis factor (TNF). This is possible by way of the extracts' ability to inhibit the NF‐κB signaling pathway, a main factor in the process of inflammation. In conclusion, the aforementioned findings suggest that this compound is the most promising material for the development of anti‐inflammatory drugs (Yuan et al. 2013). Moreover, preclinical studies utilizing animal models further support the anti‐inflammatory efficacy of *Adiantum capillus‐veneris* ; the hydro‐alcoholic extracts and the aqueous extracts from the plant were found to have a protective impact on animal models of colitis (Khoramian et al. 2020).

The reason for the introduction of these extracts is the inhibition of oxidative stress, which is mediated by the indicators myeloperoxidase (MPO) and malondialdehyde (MDA), during the course of inflammation, leading to injuries of the tissues. So, these extracts act as deterrents to leave the tissues in the same functional form and will be used for that purpose (Jalal et al. 2024; Khoramian et al. 2020). Moreover, in vivo animal studies demonstrated that the plant's bioactive compounds, notably triterpenoids and flavonoids, are effective in reducing inflammation associated with arthritis, exhibiting effects comparable to standard pharmaceutical anti‐inflammatory agents such as indomethacin (Haider et al. 2013).

The anti‐inflammatory ability of *Adiantum capillus‐veneris* is thought to come from its active ingredients, including triterpenoids and flavonoids. It has been regularly—in more than one culture—used for treating bronchitis, skin inflammations, and even gynecological issues. This has been the subject of modern phytopharmacological research, which has shown that the plant can suppress inflammatory mediators and help to repair the inflammatory tissue disorder (Jeyalatchagan Sureshkumar et al. 2018; Kimta et al. 2025). The finding of clinical tests performed as part of this research work is that the plant *Adiantum capillus‐veneris* is an effective natural anti‐inflammatory agent. It is the capacity of this plant to counteract significant inflammation mechanisms and also lessen oxidative stress that renders it a warrior resource.

### 7.5 Applications in Metabolic Disorders

Metabolic diseases are a group of diseases that are caused by the interruption of normal energy production, storage, and utilization that regulate metabolic processes. Metabolic syndrome, diabetes mellitus, obesity, and dyslipidemia are common metabolic disorders that often occur together and contribute to the increased risk for cardiovascular diseases and other complications. Among the main characteristics of metabolic syndrome are insulin resistance, hypertension, dyslipidemia, and abdominal obesity; often, it is obesity that is the leading cause of the syndrome (Eckel et al. 2005; Tune et al. 2017). Their main causes usually include mitochondrial dysfunction, oxidative stress, and systemic inflammation, which make insulin resistance worse and thus result in the development of related diseases such as nonalcoholic fatty liver disease (NAFLD) (Zheng et al. 2023).

The right lifestyle interventions, dietary changes, and physical activity lead to the prevention and management of these (Seidu et al. 2023). Bioactive compounds in plants have exhibited remarkable potential for the management of metabolic disorders such as obesity, diabetes, and metabolic syndrome. In fact, *Adiantum capillus‐veneris* , a number of diverse bioactive components, such as flavonoids, saponins, and phenolic compounds, that have been shown to be successful in metabolic health. This plant has shown hypoglycemic and even anti‐hyperlipidemic characteristics, which are critical for patients with diabetes and obese persons who suffer from metabolic disorders (Kasabri et al. 2017).

For instance, by inhibiting pancreatic lipase and alpha‐amylase, it reduced fat and glucose absorption, and then improved the lipid and glucose profiles in animal studies (Kasabri et al. 2016). Moreover, the antioxidant properties of this wonderful plant can withstand the effects of oxidation and help fight the main cause of metabolic syndrome. The neutralization of reactive species like free radicals serves as the main mechanism protecting against tissue damage and inflammation, which connects to insulin resistance and dyslipidemia (Muller et al. 2018; Roberts and Sindhu 2009). Its anti‐inflammatory activities that proceed through the NF‐κB pathway inhibition further emphasize the potential to reduce chronic inflammation associated with metabolic diseases (Baker et al. 2011). These attributes make *Adiantum capillus‐veneris* a candidate with promise for the development of complementary therapies for metabolic disorders. Further research and clinical trials are necessary to confirm its effectiveness and application in human health to its maximum level.

### 7.6 Applications as Anti‐Diabetics

Type 2 diabetes is a persistent metabolic disorder marked by elevated blood glucose levels resulting from insulin resistance and compromised pancreatic insulin production. It accounts for 90%–95% of global diabetes cases and is a major contributor to morbidity and mortality worldwide (Cantley and Ashcroft 2015). In 2019, almost 463 million persons aged 20–79 years were diagnosed with diabetes globally, with a prevalence rate of 9.3%. The figure is anticipated to rise by 25% to 578 million by 2030 and by 51% to 700 million by 2045. Diabetes is not equally distributed over all areas, as 10.8% of city populations are affected, while only 7.2% of rural populations are reported. The concentration of the disease is especially acute in wealthier countries since 10.4% belong to this group as opposed to only 4.0% in poorer nations. The actual number of undiagnosed diabetics is approximately a little less than half (50.1%) of those who suffer from the condition (Saeedi et al. 2019).

Worldwide, the occurrence of type 2 diabetes is increasing rapidly, especially in low‐ and middle‐income countries. A considerable rise in type 2 diabetes cases was recorded worldwide between 1990 and 2019, with a projected global prevalence of 10.23% in 2050. The chief factors for this are urbanization, the aging demographic, and lifestyle changes such as a sedentary way of life and obesity as a result (Liu et al. 2022). In 2019, type 2 diabetes was associated with 1.6 million deaths and significant disability‐adjusted life years (DALYs) all around the globe, showing its enormous health burden. Raised body mass index, sedentary lifestyle, and improper nutrition were the main risk factors (Yang et al. 2024; Zhang et al. 2022).

The plant extractions have been used by humans since ancient times to cure, alleviate, or relieve medical conditions (Qadir et al. 2025). Moreover, plants are considered the most scientifically attention as possible therapies for various medical conditions. Various vascular diseases, such as atherosclerosis, cerebrovascular accidents, diabetes, hypertension, and Alzheimer's disease, have all been the focus of such studies (Abdulkhader et al. 2023; Gholipour et al. 2018). *Adiantum capillus‐veneris* is known to have numerous benefits, such as aiding in the prevention of diabetes; thus, it is rightly regarded as an anti‐diabetic agent (Ranjan et al. 2014).

Further, in experimental models, *Adiantum capillus‐veneris* has demonstrated the ability to reduce blood glucose concentrations and inhibit key metabolic enzymes. Studies employing aqueous and methanolic extracts in streptozotocin‐induced diabetic rats further substantiate the ethnopharmacological use of this plant in the management of hyperglycemia (Ranjan et al. 2014; Rastogi et al. 2018). The research conducted by El Barky et al. (2017) has demonstrated that saponins act as dual‐acting agents, that is, anti‐diabetic and hypocholesterolemic (El Barky et al. 2017).

Furthermore, in another clinical model, studies on animals have also proved that terpenoids have the capability of lowering blood glucose levels (El Barky et al. 2017). Moreover, the presence of its alkaloids was clearly shown to reduce fasting blood glucose levels and to enhance kidney and liver functions in diabetic models. The healthcare field considers it an incredible option for diabetes treatment (Dizaye and Aziz 2020). *Adiantum capillus‐veneris* has the mechanism by which α‐amylase and α‐glucosidase are blocked, thus eliminating the digestion of carbohydrates and allowing for better management of blood sugar levels after a meal (Kasabri et al. 2016). Furthermore, another article revealed that the ethyl acetate fraction promotes anti‐hyperglycemic activity through inhibition of the α‐glucosidase and α‐amylase enzymes, which are the major regulators of glucose absorption (Anuradha et al. 2022).

The plant has also been noticed to be useful in the combined therapies of wound mending in diabetic animals, promoting mesenchymal growth, and neovascularization through the expression of vascular endothelial growth factor (VEGF) and transforming growth factor beta‐1 (TGF‐β1) (Negahdari et al. 2017). Research in this area shines a light on the possibility of an alternative treatment that not only aids in alleviating the disorder but also deals with the inevitable complications.

## 8 Discussion and Conclusion

*Adiantum capillus‐veneris* is a plant that is used medicinally with a rich phytochemical profile—including flavonoids, triterpenoids, saponins, and tannins—responsible for its significant antioxidant, anti‐inflammatory, antimicrobial, and anti‐diabetic activities. In vitro and preclinical studies confirm its potential to combat oxidative stress, inhibit inflammatory mediators, and suppress microbial growth, including drug‐resistant strains. These findings align with its traditional uses in treating respiratory, gastrointestinal, and inflammatory conditions (Boukada et al. 2022). Despite its broad pharmacological spectrum, several challenges hinder its clinical translation. One major limitation is the lack of standardization in extraction techniques and phytochemical profiling. Varied solvents, inconsistent methodologies, and unquantified active constituents limit reproducibility and comparability across studies. Although modern extraction methods like Soxhlet and analytical techniques such as GC–MS have improved bioactive compound isolation, a universal standard is still needed (Christou et al. 2024; Saxena 2023). Another significant gap lies in the absence of well‐designed clinical trials. Most evidence is restricted to laboratory or animal models, while clinical validation regarding efficacy, safety, and dosage in humans remains insufficient. This severely limits its acceptance in evidence‐based medicine.

Furthermore, the mechanistic understanding of its therapeutic actions is still emerging. While inhibition of NF‐κB signaling, ROS scavenging, and enzyme suppression (e.g., α‐glucosidase) have been proposed, deeper molecular insights are necessary to fully elucidate how its bioactives interact with physiological systems. Issues such as low bioavailability and stability of plant‐derived compounds also present formulation challenges. Solutions like nanoformulation, encapsulation, and biopolymer‐based delivery systems may enhance absorption, target specificity, and shelf life. In conclusion, *Adiantum capillus‐veneris* offers a powerful link between traditional medicine and modern therapeutics. Its pharmacological properties suggest potential applications in managing oxidative stress, inflammation, metabolic disorders, and antimicrobial resistance. However, future research should focus on clinical validation, standardization of extraction protocols, mechanistic studies, and pharmaceutical formulation development to unlock its full therapeutic potential. With these efforts, *Adiantum capillus‐veneris* may evolve into a safe, effective, and sustainable natural remedy for global health challenges.

## Author Contributions

**Syamand Ahmed Qadir:** writing – review and editing (equal). **Farhang Hameed Awlqadr:** investigation (equal), project administration (equal), writing – original draft (equal). **Miran Hussein Qadir:** investigation (equal), writing – original draft (equal). **Ako Mahmood Qadir:** writing – original draft (equal), writing – review and editing (equal). **Aryan Mahmood Faraj:** conceptualization (equal), funding acquisition (equal), methodology (equal). **Mohammed N. Saeed:** writing – review and editing (equal). **Salah Abdulla Salih:** investigation (equal), software (equal), supervision (equal). **Khaled Arab:** resources (equal), software (equal), writing – review and editing (equal). **Seyed Mohammad Najibi Hosseini:** writing – review and editing (equal).

## Ethics Statement

The authors have nothing to report.

## Consent

The authors have nothing to report.

## Conflicts of Interest

The authors declare no conflicts of interest.

## Data Availability

The data that support the findings of this study are available from the corresponding author upon reasonable request.

## References

[fsn371118-bib-0001] Abdulkhader, J. , A. Ummer , S. Kareem , et al. 2023. “Potential Role of Medicinal Plants Against Alzheimer's Disease: An Update on Therapeutic Interventions.” Journal of Pharmacy and Allied Medicine 1: 42–56.

[fsn371118-bib-0002] Ahmadpoor, J. , S. V. Chahardahcheric , and M. Setorki . 2019. “The Protective Effect of Hydroalcoholic Extract of the Southern Maidenhair Fern (*Adiantum Capillus‐Veneris*) on the Depression and Anxiety Caused by Chronic Stress in Adult Male Mice: An Experimental Randomized Study.” Iranian Red Crescent Medical Journal 21, no. 3: e86750.

[fsn371118-bib-0003] Ahmadpouri, J. , S. V. Chahardahcharic , and M. Setorki . 2020. “The Effect of *Adiantum Capillus‐veneris* L. Hydroalcoholic Extract on the Oxidative Stress Rate of Mice's Blood and Brain in the Depression Model Caused by Acute Immobilization Stress.” Pharmaceutical and Biomedical Research 6, no. 2: 3803.

[fsn371118-bib-0004] Ahmed, A. , N. Jahan , A. Wadud , H. Imam , S. Hajera , and A. Bilal . 2012. “Physicochemical and Biological Properties of *Adiantum capillus‐veneris* Linn: An Important Drug of Unani System of Medicine.” International Journal of Current Research and Review 4, no. 21: 70–75.

[fsn371118-bib-0005] Ahmed, A. U. 2011. “An Overview of Inflammation: Mechanism and Consequences.” Frontiers in Biology 6, no. 4: 274–281.

[fsn371118-bib-0006] Akabori, Y. , and M. Hasegawa . 1969. “Flavonoid Pattern in the Pteridaceae II Flavonoid Constituents in the Fronds of *Adiantum Capillus‐Veneris* and *A. Cuneatum* .” Shokubutsugaku Zasshi 82, no. 973: 294–297.

[fsn371118-bib-0007] Akbar, S. , and S. Akbar . 2020. “Adiantum Venustum G. Don. / Adiantum Capillusveneris L. (Pteridaceae).” In Handbook of 200 Medicinal Plants: A Comprehensive Review of Their Traditional Medical Uses and Scientific Justifications, 103–107. Springer. 10.1007/978-3-030-16807-0.

[fsn371118-bib-0008] Al‐Hallaq, E. K. , S. C. Litescu , V. Kasabri , K. K. Abdul‐Razzak , and F. Afifi . 2015. “Hypocholesterolemic Effects of *Adiantum Capillus Veneris* L. Aqueous Extract in High Cholesterol Diet‐Fed Rats and HPLC‐MS Determination of Its Polyphenolics.” Revue Roumaine de Chimie 60: 357–365.

[fsn371118-bib-0009] Al‐Snafi, A. E. 2015a. “The Chemical Constituents and Pharmacological Effects of *Adiantum capillus‐veneris* ‐A Review.” Asian Journal of Pharmaceutical Science and Technology 5, no. 2: 106–111.

[fsn371118-bib-0010] Al‐Snafi, A. E. 2015b. Encyclopedia of the constituents and pharmacological effects of Iraqi medicinal plants. Rigi Publication.

[fsn371118-bib-0011] Anderson, O. R. 2023. “Photophysiology, Dark Respiration and Leaf Desiccation Resilience of the Fern *Adiantum Capillus‐Veneris* L.” Journal of Ecology and the Natural Environment 15: 1–8.

[fsn371118-bib-0012] Anuradha, S. , D. Mahesh Kumar , and V. Swaroopa Rani . 2022. “In Vitro Studies on α‐Amylase and α‐Glucosidase Inhibitory Activity of *Antigonon Leptopus* (Hook et. Arn) Leaf Extract and Its Fractions.” International Journal of Zoological Investigations 8: 67–74.

[fsn371118-bib-0013] Baba, S. A. , and S. A. Malik . 2015. “Determination of Total Phenolic and Flavonoid Content, Antimicrobial and Antioxidant Activity of a Root Extract of *Arisaema Jacquemontii Blume* .” Journal of Taibah University for Science 9, no. 4: 449–454.

[fsn371118-bib-0014] Baker, R. G. , M. S. Hayden , and S. K. Ghosh . 2011. “NF‐κB, Inflammation, and Metabolic Disease.” Cell Metabolism 13, no. 1: 11–22. 21195345 10.1016/j.cmet.2010.12.008 PMC3040418

[fsn371118-bib-0015] Benabderrahim, M. A. , Y. Yahia , I. Bettaieb , W. Elfalleh , and K. Nagaz . 2019. “Antioxidant Activity and Phenolic Profile of a Collection of Medicinal Plants From Tunisian Arid and Saharan Regions.” Industrial Crops and Products 138: 111427.

[fsn371118-bib-0016] Boukada, F. , S. Sitayeb , H. Khadem , B. Meddah , and F. Z. Soltani . 2022. “Chemical Composition, Antioxidant and Antibacterial Activity of *Adiantum Capillus‐Veneris* L. Extract From Algeria.” Kragujevac Journal of Science 44: 4091.

[fsn371118-bib-0017] Brenner, P. , and T. B. S. P. Krakauer . 2003. “Regulation of Inflammation: A Review of Recent Advances in Anti‐ Inflammatory Strategies.” Current Medicinal Chemistry: Anti‐Inflammatory & Anti‐Allergy Agents 2: 274–283.

[fsn371118-bib-0018] Caballero‐Sánchez, N. , S. Alonso‐Alonso , and L. Nagy . 2024. “Regenerative Inflammation: When Immune Cells Help to Re‐Build Tissues.” FEBS Journal 291, no. 8: 1597–1614. 36440547 10.1111/febs.16693 PMC10225019

[fsn371118-bib-0019] Cantley, J. , and F. M. Ashcroft . 2015. “Q&A: Insulin Secretion and Type 2 Diabetes: Why Do β‐Cells Fail?” BMC Biology 13: 33. 25982967 10.1186/s12915-015-0140-6 PMC4435650

[fsn371118-bib-0020] Cappiello, F. , M. R. Loffredo , C. Del Plato , et al. 2020. “The Revaluation of Plant‐Derived Terpenes to Fight Antibiotic‐Resistant Infections.” Antibiotics 9, no. 6: 325. 32545761 10.3390/antibiotics9060325 PMC7344648

[fsn371118-bib-0021] Chauhan, S. K. , R. Sahu , and L. S. B. Upadhyay . 2019. “Medicinal Plants: A Potent Antimicrobial Source and An Alternative to Combat Antibiotic Resistance.” In Ethnopharmacology and Biodiversity of Medicinal Plants, edited by A. A. Alqarawi , C. M. Abdel‐Latif , and M. Ahmad , 239–264. Apple Academic Press, CRC Press (Taylor & Francis Group).

[fsn371118-bib-0022] Chemat, F. , and J. Strube . 2015. Green Extraction of Natural Products: Theory and Practice. John Wiley & Sons.

[fsn371118-bib-0023] Chen, L. , H. Deng , H. Cui , et al. 2015. “Inflammatory Responses and Inflammation‐Associated Diseases in Organs.” Oncotarget 9: 7204–7218. 10.18632/oncotarget.23208 PMC5805548 29467962

[fsn371118-bib-0024] Christou, A. , C. Stavrou , C. Michael , G. Botsaris , and V. Goulas . 2024. “New Insights Into the Potential Inhibitory Effects of Native Plants From Cyprus on Pathogenic Bacteria and Diabetes‐Related Enzymes.” Microbiology Research 15: 926–942.

[fsn371118-bib-0025] Dalamagka, M. I. 2024. “Integrating Traditional Medicine Into a Modern Health Care System.” International Journal of Science and Research Archive 12, no. 1: 2372–2375.

[fsn371118-bib-0026] Dalli, J. , and C. N. Serhan . 2018. “Identification and Structure Elucidation of the Pro‐Resolving Mediators Provides Novel Leads for Resolution Pharmacology.” British Journal of Pharmacology 176: 1024–1037. 29679485 10.1111/bph.14336 PMC6451074

[fsn371118-bib-0027] Daskou, M. , L. Fotooh Abadi , C. Gain , et al. 2023. “The Role of the NRF2 Pathway in the Pathogenesis of Viral Respiratory Infections.” Pathogens 13: 39. 38251346 10.3390/pathogens13010039 PMC10819673

[fsn371118-bib-0028] Dehdari, S. , and H. Hajimehdipoor . 2018. “Medicinal Properties of *Adiantum Capillus‐Veneris* Linn. in Traditional Medicine and Modern Phytotherapy: A Review Article.” Iranian Journal of Public Health 47, no. 2: 188–197. 29445628 PMC5810381

[fsn371118-bib-0029] Dizaye, K. F. , and R. S. Aziz . 2020. “Antihyperglycemic effect of the alkaloids extracted from *Adiantum capillus* in diabetic rats.” Ann Coll Med Mosul 41, no. 2: 148–157.

[fsn371118-bib-0030] Eckel, R. H. , S. M. Grundy , and P. Z. Zimmet . 2005. “The Metabolic Syndrome.” Lancet 365, no. 9468: 1415–1428. 15836891 10.1016/S0140-6736(05)66378-7

[fsn371118-bib-0031] El Barky, A. R. , S. A. Hussein , A. Alm‐Eldeen , Y. A. Hafez , and T. M. Mohamed . 2017. “Saponins and Their Potential Role in Diabetes Mellitus.” Diabetes Manag 7, no. 1: 148–158.

[fsn371118-bib-0032] Farràs, A. , V. López , F. Maggi , G. Caprioli , M. P. Vinardell , and M. Mitjans . 2022. “Chemical Composition and Cytoprotective Activities of Methanolic Extract of *Asplenium Adiantum‐Nigrum* L.(Aspleniaceae).” Horticulturae 8, no. 9: 815.

[fsn371118-bib-0033] Foo, J. C. H. , G. L. Bellot , S. Pervaiz , and S. Alonso . 2022. “Mitochondria‐Mediated Oxidative Stress During Viral Infection.” Trends in Microbiology 30: 679–692. 35063304 10.1016/j.tim.2021.12.011

[fsn371118-bib-0034] Fratta Pasini, A. M. , C. Stranieri , L. Cominacini , and C. Mozzini . 2021. “Potential Role of Antioxidant and Anti‐Inflammatory Therapies to Prevent Severe SARS‐Cov‐2 Complications.” Antioxidants 10: 272. 33578849 10.3390/antiox10020272 PMC7916604

[fsn371118-bib-0035] Garofalo, R. P. , D. Kolli , and A. Casola . 2013. “Respiratory Syncytial Virus Infection: Mechanisms of Redox Control and Novel Therapeutic Opportunities.” Antioxidants & Redox Signaling 18, no. 2: 186–217. 22799599 10.1089/ars.2011.4307 PMC3513983

[fsn371118-bib-0036] Gholipour, S. , R. D. E. Sewell , Z. Lorigooini , and M. Rafieian‐kopaei . 2018. “Medicinal Plants and Atherosclerosis: A Review on Molecular Aspects.” Current Pharmaceutical Design 24, no. 26: 3123–3131. 30205790 10.2174/1381612824666180911121525

[fsn371118-bib-0037] Ghosh, M.,. R. , and K. Gupta . 2005. “Antifungal Activity of the Crude Extracts and Extracted Phenols from Gametophytes and Sporophytes of Two Species of Adiantum.” Taiwania 50: 272–283.

[fsn371118-bib-0038] Gorlenko, C. L. , H. Y. Kiselev , E. V. Budanova , A. A. Zamyatnin , and L. N. Ikryannikova . 2020. “Plant Secondary Metabolites in the Battle of Drugs and Drug‐Resistant Bacteria: New Heroes or Worse Clones of Antibiotics?” Antibiotics 9: 170. 32290036 10.3390/antibiotics9040170 PMC7235868

[fsn371118-bib-0039] Gülcin, I. 2012. “Antioxidant Activity of Food Constituents: an Overview.” Archives of Toxicology 86: 345–391. 22102161 10.1007/s00204-011-0774-2

[fsn371118-bib-0040] Hadi, I. , and H. M. Hussein . 2016. “Antimicrobial Activity and Spectral Chemical Analysis of Methanolic Leaves Extract of *Adiantum Capillus‐Veneris* Using GC‐MS and FT‐IR Spectroscopy.” International Journal of Pharmacognosy and Phytochemical Research 8, no. 3: 369–385.

[fsn371118-bib-0041] Haider, S. , C. Kharbanda , M. S. Alam , et al. 2013. “Anti‐Inflammatory and Anti‐Nociceptive Activities of Two New Triterpenoids From *Adiantum Capillus‐Veneris* Linn.” Natural Product Research 27: 2304–2310. 23972143 10.1080/14786419.2013.828292

[fsn371118-bib-0042] Ham, H. , K. S. Woo , B.‐Z. Lee , et al. 2015. “Antioxidant Compounds and Activities of Methanolic Extracts From Oat Cultivars.” Journal of the Korean Society of Food Science and Nutrition 44: 1660–1665.

[fsn371118-bib-0043] Herzyk, F. , D. Piłakowska‐Pietras , and M. Korzeniowska . 2024. “Supercritical Extraction Techniques for Obtaining Biologically Active Substances From a Variety of Plant Byproducts.” Food 13: 1713. 10.3390/foods13111713 PMC11171758 38890941

[fsn371118-bib-0044] Ibourki, M. , O. Hallouch , K. P. Devkota , D. Guillaume , A. Hirich , and S. Gharby . 2023. “Elemental Analysis in Food: An Overview.” Journal of Food Composition and Analysis 120: 105330.

[fsn371118-bib-0045] Ibraheim, Z. Z. , A. Ahmed , and Y. G. Gouda . 2011. “Phytochemical and Biological Studies of *Adiantum capillus‐veneris* L.” Saudi Pharmaceutical Journal : SPJ : The Official Publication of the Saudi Pharmaceutical Society 19, no. 2: 65–74. 23960744 10.1016/j.jsps.2011.01.007 PMC3745073

[fsn371118-bib-0046] Ishaq, M. S. , M. M. Hussain , M. S. Afridi , et al. 2014. “In Vitro Phytochemical, Antibacterial, and Antifungal Activities of Leaf, Stem, and Root Extracts of *Adiantum Capillus Veneris* .” Scientific World Journal 2014: 269793. 24592156 10.1155/2014/269793 PMC3925560

[fsn371118-bib-0047] Jaafer, M. , W. Mahood , and M. Alyami . 2025. “ *Adiantum Capillus‐veneris* L.: A Comprehensive Review of Its Phytochemical Composition, Pharmacological Activities, and Therapeutic Potential.” Baghdad Journal of Biochemistry and Applied Biological Sciences 6, no. 2: 85–99.

[fsn371118-bib-0048] Jalal, S. , S. Fakhri , M. R. Morovati , E. Mohammadi Noori , S. Miraghaee , and M. H. Farzaei . 2024. “Evaluation of the Protective Effect of Hydroalcoholic and Aqueous Extracts of *Ulmus minor* Leaves on Ulcerative Colitis Induced by Acetic Acid in Rats.” Jundishapur Journal of Natural Pharmaceutical Products 19: e142668.

[fsn371118-bib-0049] Jansen, C. , J. D. Baker , E. Kodaira , et al. 2020. “Medicine in Motion: Opportunities, Challenges and Data Analytics‐Based Solutions for Traditional Medicine Integration Into Western Medical Practice.” Journal of Ethnopharmacology 267: 113477. 33098971 10.1016/j.jep.2020.113477 PMC7577282

[fsn371118-bib-0050] Jeyalatchagan Sureshkumar, J. S. , R. S. Rajendran Silambarasan , K. Bharati , J. K. Jayaraj Krupa , S. A. Singamoorthy Amalraj , and M. A. Muniappan Ayyanar . 2018. “A Review on Ethnomedicinally Important Pteridophytes of India.” A Review on Ethnomedicinally Important Pteridophytes of India 219: 269–287. 10.1016/j.jep.2018.03.024 29578072

[fsn371118-bib-0051] Jiang, M.‐Z. , H. Yan , Y. Wen , and X.‐M. Li . 2011. “In Vitro and In Vivo Studies of Antioxidant Activities of Flavonoids From *Adiantum capillus‐veneris* L.” African Journal of Pharmacy and Pharmacology 5, no. 18: 2079–2085.

[fsn371118-bib-0052] Jin, Y. , and R. Arroo . 2023. “The Protective Effects of Flavonoids and Carotenoids Against Diabetic Complications—A Review of In Vivo Evidence.” Frontiers in Nutrition 10: 1020950. 37032781 10.3389/fnut.2023.1020950 PMC10080163

[fsn371118-bib-0054] Jones, D. P. 2006. “Redefining Oxidative Stress.” Antioxidants & Redox Signaling 8, no. 9–10: 1865–1879. 16987039 10.1089/ars.2006.8.1865

[fsn371118-bib-0053] Jones, D. P. 2008. “Radical‐Free Biology of Oxidative Stress.” American Journal of Physiology. Cell Physiology 295, no. 4: C849–C868. 18684987 10.1152/ajpcell.00283.2008 PMC2575825

[fsn371118-bib-0055] Juan, C. A. , J. M. Pérez de la Lastra , F. J. Plou , and E. Perez‐Lebeña . 2021. “The Chemistry of Reactive Oxygen Species (ROS) Revisited: Outlining Their Role in Biological Macromolecules (DNA, Lipids and Proteins) and Induced Pathologies.” International Journal of Molecular Sciences 22: 4642. 33924958 10.3390/ijms22094642 PMC8125527

[fsn371118-bib-0056] Kasabri, V. , E. K. Al‐Hallaq , Y. K. Bustanji , K. K. Abdul‐Razzak , I. F. Abaza , and F. U. Afifi . 2016. “Antiobesity and Antihyperglycaemic Effects of *Adiantum Capillus‐Veneris* Extracts: In Vitro and In Vivo Evaluations.” Pharmaceutical Biology 55: 164–172. 27663206 10.1080/13880209.2016.1233567 PMC7011982

[fsn371118-bib-0057] Kashkooe, A. , F. A. Sardari , M. M. Mehrabadi , and M. M. Zarshenas . 2020. “A Review on Pharmacological Properties and Toxicological Effects of *Adiantum capillus‐veneris* L.” Current Drug Discovery Technologies 18, no. 2: 186–193. 10.2174/1570163817666200316111445 32178614

[fsn371118-bib-0058] Khodaie, L. , S. Esnaashari , and S. B. Moghaddam . 2015. “Essential Oil of Arial Parts of *Adiantum Capillus‐Veneris* : Chemical Composition and Antioxidant Activity.” Jundishapur Journal of Natural Pharmaceutical Products 10: e21968.

[fsn371118-bib-0059] Khomich, O. , S. N. Kochetkov , B. Bartosch , and A. V. Ivanov . 2018. “Redox Biology of Respiratory Viral Infections.” Viruses 10: 392. 30049972 10.3390/v10080392 PMC6115776

[fsn371118-bib-0060] Khoramian, L. , S.‐E. Sajjadi , and M. Minaiyan . 2020. “Anti‐Inflammatory Effect of *Adiantum capillus‐veneris* Hydroalcoholic and Aqueous Extracts on Acetic Acid‐Induced Colitis in Rats.” Avicenna Journal of Phytomedicine 10, no. 5: 492–503. 32995327 PMC7508316

[fsn371118-bib-0061] Kimta, N. , S. Puri , A. Kumari , et al. 2025. “Applications of Pteridophytes in Nanotechnology: A Class That Has Not Yet Explored to the Extent of Its Potential.” Green Chemistry Letters and Reviews 18, no. 1: 2460641.

[fsn371118-bib-0062] Komaravelli, N. , and A. Casola . 2014. “Respiratory Viral Infections and Subversion of Cellular Antioxidant Defenses.” Journal of Pharmacogenomics & Pharmacoproteomic 5: 1000141. 10.4172/2153-0645.1000141 PMC4288774 25584194

[fsn371118-bib-0063] Kumar, V. , V. Charde , S. B. Prasad , et al. 2023. “Therapeutic Potential of Evergreen Maiden Hair Fern *Adiantum Venustum* D. Don: A Comprehensive Review.” Food Chemistry Advances 3: 100439.

[fsn371118-bib-0064] Lakshmi, K. M. R. , M. Kiran , and K. S. Prasanna . 2021. “A Review on Natural Plants for Phytochemical Constituents and Pharmacological Activities.” Journal of Drug Delivery and Therapeutics 11: 232–236.

[fsn371118-bib-0065] Libby, P. 2007. “Inflammatory Mechanisms: the Molecular Basis of Inflammation and Disease.” Nutrition Reviews 65: S140–S146. 18240538 10.1111/j.1753-4887.2007.tb00352.x

[fsn371118-bib-0066] Liu, J. , R. Bai , Z. Chai , M. E. Cooper , P. Z. Zimmet , and L. Zhang . 2022. “Low‐ and Middle‐Income Countries Demonstrate Rapid Growth of Type 2 Diabetes: An Analysis Based on Global Burden of Disease 1990–2019 Data.” Diabetologia 65: 1339–1352. 35587275 10.1007/s00125-022-05713-6 PMC9118183

[fsn371118-bib-0067] López‐Salazar, H. , B. H. Camacho‐Díaz , M. A. Ocampo , and A. R. Jiménez‐Aparicio . 2023. “Microwave‐Assisted Extraction of Functional Compounds From Plants: A Review.” BioResources 18, no. 3: 6614–6638.

[fsn371118-bib-0068] Madhab, M. , C. Mangla , S. Vijaya , et al. 2023. “Different Biological Activities Especially Antioxidant Activity of Plant‐Based Functional Foods for Human Health.” International Journal of Membrane Science and Technology 10, no. 4: 2419–2423.

[fsn371118-bib-0069] Manisha, D. R. B. , A. M. Begam , K. S. Chahal , and M. A. Ashok . 2025. “Medicinal Plants and Traditional Uses and Modern Applications.” Journal of Neonatal Surgery 14, no. 3: 2210.

[fsn371118-bib-0070] Megha, K. , X. Joseph , V. P. Akhil , and P. V. Mohanan . 2021. “Cascade of Immune Mechanism and Consequences of Inflammatory Disorders.” Phytomedicine 91: 153712. 34511264 10.1016/j.phymed.2021.153712 PMC8373857

[fsn371118-bib-0071] Mickel, J. T. 2004. “The Pteridophytes of Mexico.” Memoirs of the New York Botanical Garden 88: 1.

[fsn371118-bib-0072] Moghimipour, E. , and S. Handali . 2015. “Saponin: Properties, Methods of Evaluation and Applications.” Annual Research & Review in Biology 5, no. 3: 207–220.

[fsn371118-bib-0073] Muller, C. J. F. , C. J. Malherbe , N. Chellan , K. Yagasaki , Y. Miura , and E. Joubert . 2018. “Potential of Rooibos, Its Major C‐Glucosyl Flavonoids, and Z‐2‐(β‐D‐Glucopyranosyloxy)‐3‐Phenylpropenoic Acid in Prevention of Metabolic Syndrome.” Critical Reviews in Food Science and Nutrition 58: 227–246. 27305453 10.1080/10408398.2016.1157568

[fsn371118-bib-0074] Murakami, M. , and T. Hirano . 2012. “The Molecular Mechanisms of Chronic Inflammation Development.” Frontiers in Immunology 3: 323. 23162547 10.3389/fimmu.2012.00323 PMC3498841

[fsn371118-bib-0075] Naqishbandi, A. 2014. “Plants Used in Iraqi Traditional Medicine in Erbil‐Kurdistan Region.” Zanco Journal of Medical Sciences (Zanco J Med Sci) 18, no. 3: 811–815.

[fsn371118-bib-0076] Nasrollahi, I. , E. Talebi , and Z. Bashardoost . 2022. “In‐vitro Study of Chemical Composition, Antimicrobial and Antioxidant Properties of Adiantum capillus‐veneris L.” Essential Oil. Preprints: 2022070214. 10.20944/preprints202207.0214.v1.

[fsn371118-bib-0077] Negahdari, S. , H. Galehdari , M. Kesmati , A. Rezaie , G. Shariati , and M. S. Shapouri . 2017. “Analysis of VEGF AND TGFB1 Proteins in Normal and Streptozotocin‐Induced Diabetic Rats, During Treatment of Formulations of *Aloe Vera*, Henna, *Adiantum Capillus‐Veneris*, and Myrrha.” Wound Medicine 16: 22–28.

[fsn371118-bib-0078] Parham, S. , A. Z. Kharazi , H. R. Bakhsheshi‐Rad , et al. 2020. “Antioxidant, Antimicrobial and Antiviral Properties of Herbal Materials.” Antioxidants 9: 1309. 33371338 10.3390/antiox9121309 PMC7767362

[fsn371118-bib-0079] Pheko‐Ofitlhile, T. , and A. Makhzoum . 2024. “Impact of Hydrodistillation and Steam Distillation on the Yield and Chemical Composition of Essential Oils and Their Comparison With Modern Isolation Techniques.” Journal of Essential Oil Research 36: 105–115.

[fsn371118-bib-0080] Pohl, P. , A. Dzimitrowicz , D. Jedryczko , A. Szymczycha‐Madeja , M. Wełna , and P. Jamróz . 2016. “The Determination of Elements in Herbal Teas and Medicinal Plant Formulations and Their Tisanes.” Journal of Pharmaceutical and Biomedical Analysis 130: 326–335. 26830083 10.1016/j.jpba.2016.01.042

[fsn371118-bib-0081] Qadir, S. A. , F. H. Awlqadr , M. H. Qadir , et al. 2025. “Bioactivities, Medicinal Properties, and Advanced Extraction Techniques of Tarragon (*Artemisia Dracunculus*): A Comprehensive Review.” Journal of Herbal Medicine 50: 100989.

[fsn371118-bib-0082] Rabiei, Z. , and M. Setorki . 2019. “Effect of Ethanol *Adiantum Capillus‐Veneris* Extract in Experimental Models of Anxiety and Depression.” Brazilian Journal of Pharmaceutical Sciences 55: e18099.

[fsn371118-bib-0083] Rajurkar, N. , and K. Gaikwad . 2012. “Evaluation of Phytochemicals, Antioxidant Activity and Elemental Content of *Adiantum Capillus Veneris* Leaves.” Journal of Chemical and Pharmaceutical Research 4, no. 1: 365–374.

[fsn371118-bib-0084] Rakesh, S. U. , P. R. Patil , and S. R. Mane . 2010. “Use of Natural Antioxidants to Scavenge Free Radicals: a Major Cause of Diseases.” International Journal of PharmTech Research 2: 1074–1081.

[fsn371118-bib-0085] Ranjan, V. , M. Vats , N. Gupta , and S. Sardana . 2014. “Antidiabetic Potential of Whole Plant of *Adiantum capillus veneris* Linn. in Streptozotocin Induced Diabetic Rats.” International Journal of Pharmaceutical and Clinical Research 6, no. 4: 341–347.

[fsn371118-bib-0086] Rastogi, S. , M. M. Pandey , and A. K. S. Rawat . 2018. “Ethnopharmacological Uses, Phytochemistry and Pharmacology of Genus Adiantum: A Comprehensive Review.” Journal of Ethnopharmacology 215: 101–119. 29288826 10.1016/j.jep.2017.12.034

[fsn371118-bib-0087] Ray, P. D. , B.‐W. Huang , and Y. Tsuji . 2012. “Reactive Oxygen Species (ROS) Homeostasis and Redox Regulation in Cellular Signaling.” Cellular Signalling 24, no. 5: 981–990. 22286106 10.1016/j.cellsig.2012.01.008 PMC3454471

[fsn371118-bib-0088] Roberts, C. K. , and K. K. Sindhu . 2009. “Oxidative Stress and Metabolic Syndrome.” Life Sciences 84, no. 21–22: 705–712. 19281826 10.1016/j.lfs.2009.02.026

[fsn371118-bib-0089] Roy, S. , B. Hazra , N. Mandal , and T. K. Chaudhuri . 2013. “Assessment of the Antioxidant and Free Radical Scavenging Activities of Methanolic Extract of *Diplazium Esculentum* .” International Journal of Food Properties 16, no. 6: 1351–1370.

[fsn371118-bib-0090] Roy, S. C. , B. Sajeeb , A. Muhit , and S. C. Bachar . 2019. “Evaluation of Antioxidant and Cytotoxic Activities of Aerial Parts of *Adiantum Capillus‐Veneris* L. Growing in Bangladesh.” Dhaka University Journal of Pharmaceutical Sciences 18: 217–222.

[fsn371118-bib-0091] Saeedi, P. , I. Petersohn , P. Salpea , et al. 2019. “Global and Regional Diabetes Prevalence Estimates for 2019 and Projections for 2030 and 2045: Results From the International Diabetes Federation Diabetes Atlas, 9th Edition.” Diabetes Research and Clinical Practice 157: 107843. 31518657 10.1016/j.diabres.2019.107843

[fsn371118-bib-0092] Santos, C. X. C. , S. Raza , and A. M. Shah . 2016. “Redox Signaling in the Cardiomyocyte: From Physiology to Failure.” International Journal of Biochemistry & Cell Biology 74: 145–151. 26987585 10.1016/j.biocel.2016.03.002

[fsn371118-bib-0093] Sapoliya, N. K. , and M. B. Shah . 2022. “Regulations on Herbal Products in India, United States and European Union: A Review.” International Journal of Drug Regulatory Affairs 10, no. 2: 67–72.

[fsn371118-bib-0094] Satoh, K. , P. Nigro , and B. C. Berk . 2010. “Oxidative Stress and Vascular Smooth Muscle Cell Growth: A Mechanistic Linkage by Cyclophilin A.” Antioxidants & Redox Signaling 12, no. 5: 675–682. 19747062 10.1089/ars.2009.2875 PMC2861539

[fsn371118-bib-0095] Saxena, R. 2023. “Exploring Approaches for Investigating Phytochemistry: Methods and Techniques.” Medalion journal: Medical Research, Nursing, Health and Midwife Participation 4, no. 2: 65–73.

[fsn371118-bib-0096] Schieber, M. , and N. S. Chandel . 2014. “ROS Function in Redox Signaling and Oxidative Stress.” Current Biology 24: R453–R462. 24845678 10.1016/j.cub.2014.03.034 PMC4055301

[fsn371118-bib-0097] Schwartz, M. 2012. Adiantum Capillus‐Veneris L.: Ethnobotany, Etymology, and Iranian Cultural History. Vol. 26, 97–101. Bulletin of the Asia Institute.

[fsn371118-bib-0098] Seidu, B. S. , H. Osman , and S. Seidu . 2023. “Lifestyle or Pharmacotherapy in Cardio‐Metabolic Disease Prevention.” Therapeutic Advances in Cardiovascular Disease 17: 17539447231177175. 37381922 10.1177/17539447231177175 PMC10334018

[fsn371118-bib-0099] Shen, L. , S. Pang , M. Zhong , et al. 2023. “A Comprehensive Review of Ultrasonic Assisted Extraction (UAE) for Bioactive Components: Principles, Advantages, Equipment, and Combined Technologies.” Ultrasonics Sonochemistry 101: 106646. 37862945 10.1016/j.ultsonch.2023.106646 PMC10594638

[fsn371118-bib-0100] Singh, M. , N. Singh , P. Khare , and A. K. S. Rawat . 2008. “Antimicrobial Activity of Some Important *Adiantum* Species Used Traditionally in Indigenous Systems of Medicine.” Journal of Ethnopharmacology 115, no. 2: 327–329. 17997240 10.1016/j.jep.2007.09.018

[fsn371118-bib-0101] Talebi, E. , I. Nasrollahi , and Z. Bashardoost . 2022. “Phytochemical Compounds and Bioactivity Properties of the Whole Plant of Maidenhair Fern (*Adiantum Capillus‐Veneris* L.) Essential Oil.” Safe Future and Agricultural Research Journal (SFARJ) 1, no. 1: 1–10.

[fsn371118-bib-0102] Tune, J. D. , A. G. Goodwill , D. J. Sassoon , and K. J. Mather . 2017. “Cardiovascular Consequences of Metabolic Syndrome.” Translational Research : The Journal of Laboratory and Clinical Medicine 183: 57–70. 28130064 10.1016/j.trsl.2017.01.001 PMC5393930

[fsn371118-bib-0103] Vadi, R. , V. Manisha , and K. Swati . 2017. “Hansraj (*Adiantum Capillus Veneris* Linn.): A Systematic Review on Its Ethnobotany, Phytochemical and Pharmacological Profile.” International Journal of Ayurveda and Pharma Research 5: 5–21.

[fsn371118-bib-0104] Vardar Acar, N. , and R. K. Özgül . 2023. “The Bridge Between Cell Survival and Cell Death: Reactive Oxygen Species‐Mediated Cellular Stress.” EXCLI Journal 22: 520–555. 37534225 10.17179/excli2023-6221 PMC10390897

[fsn371118-bib-0105] Victor, B. , M. Maridass , U. Ramesh , and J. Prabhu . 1970. “Antibacterial Activity of Essential Oils From the Leaves of *Adiantum Capillus Veneris* Linn.” Malaysian Journal of Science 22: 65–66.

[fsn371118-bib-0106] Xu, Q. , L. P. Huff , M. Fujii , and K. K. Griendling . 2017. “Redox Regulation of the Actin Cytoskeleton and Its Role in the Vascular System.” Free Radical Biology and Medicine 109: 84–107. 28285002 10.1016/j.freeradbiomed.2017.03.004 PMC5497502

[fsn371118-bib-0107] Yadav, A. , and V. N. Dange . 2020. “Mechanism Involved in the Process of Inflammation.” International Journal of Pharmacology and Pharmaceutical Sciences 2, no. 1: 1–6.

[fsn371118-bib-0108] Yadav, S. 2021. “Potential Role of Medicinal Plants Against Alzheimer's Disease.” International Journal of Complementary & Alternative Medicine 14: 138–140.

[fsn371118-bib-0109] Yang, X. , J. Sun , and W. Zhang . 2024. “Global Trends in Burden of Type 2 Diabetes Attributable to Physical Inactivity Across 204 Countries and Territories, 1990‐2019.” Frontiers in Endocrinology 15: 1343002. 38469145 10.3389/fendo.2024.1343002 PMC10925666

[fsn371118-bib-0110] Yuan, Q. , J. Wang , and J. Ruan . 2012. “Screening for Bioactive Compounds From *Adiantum Capillus–Veneris* L.” Journal of the Chemical Society of Pakistan 34, no. 1: 207–216.

[fsn371118-bib-0111] Yuan, Q. , X. Zhang , Z. Liu , et al. 2013. “Ethanol Extract of *Adiantum Capillus‐Veneris* L. Suppresses the Production of Inflammatory Mediators by Inhibiting NF‐κB Activation.” Journal of Ethnopharmacology 147, no. 3: 603–611. 23542147 10.1016/j.jep.2013.03.046

[fsn371118-bib-0112] Yung, L. M. , F. P. Leung , X. Yao , Z.‐Y. Chen , and Y. Huang . 2006. “Reactive Oxygen Species in Vascular Wall.” Cardiovascular & Hematological Disorders Drug Targets 6, no. 1: 1–19. 16724932 10.2174/187152906776092659

[fsn371118-bib-0113] Zeb, A. , and F. Ullah . 2017. “Reversed Phase HPLC‐DAD Profiling of Carotenoids, Chlorophylls and Phenolic Compounds in *Adiantum Capillus‐Veneris* Leaves.” Frontiers in Chemistry 5: 29. 28497036 10.3389/fchem.2017.00029 PMC5406511

[fsn371118-bib-0114] Zhang, X. , X. Wang , M.‐r. Wang , et al. 2022. “The Global Burden of Type 2 Diabetes Attributable to High Body Mass Index in 204 Countries and Territories, 1990–2019: An Analysis of the Global Burden of Disease Study.” Frontiers in Public Health 10: 966093. 36159296 10.3389/fpubh.2022.966093 PMC9500174

[fsn371118-bib-0115] Zheng, Y. , S. Wang , J. Wu , and Y. Wang . 2023. “Mitochondrial Metabolic Dysfunction and Non‐Alcoholic Fatty Liver Disease: New Insights From Pathogenic Mechanisms to Clinically Targeted Therapy.” Journal of Translational Medicine 21: 510. 37507803 10.1186/s12967-023-04367-1 PMC10375703

